# Willow Bark-Derived Material with Antibacterial and Antibiofilm Properties for Potential Wound Dressing Applications

**DOI:** 10.1021/acs.jafc.3c00849

**Published:** 2023-04-27

**Authors:** Jinze Dou, Polina Ilina, Cristina D. Cruz, Denise Nurmi, Paula Zegarra Vidarte, Marja Rissanen, Päivi Tammela, Tapani Vuorinen

**Affiliations:** † Department of Bioproducts and Biosystems, School of Chemical Engineering, Aalto University, 00076 Aalto, Finland; ‡ Drug Research Program, Division of Pharmaceutical Biosciences, Faculty of Pharmacy, University of Helsinki, 00014 Helsinki, Finland

**Keywords:** chemical structure−property relationships, lignin, unsaturated fatty acids, wound dressing, bark biorefinery, willow bark fiber bundle, antibacterial, antibiofilm, *Staphylococcus aureus*

## Abstract

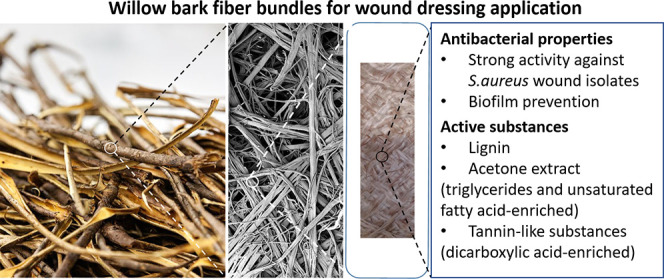

Tree stems contain wood in addition to 10–20% bark, which remains one of the largest underutilized biomasses on earth. Unique macromolecules (like lignin, suberin, pectin, and tannin), extractives, and sclerenchyma fibers form the main part of the bark. Here, we perform detailed investigation of antibacterial and antibiofilm properties of bark-derived fiber bundles and discuss their potential application as wound dressing for treatment of infected chronic wounds. We show that the yarns containing at least 50% of willow bark fiber bundles significantly inhibit biofilm formation by wound-isolated *Staphylococcus aureus* strains. We then correlate antibacterial effects of the material to its chemical composition. Lignin plays the major role in antibacterial activity against planktonic bacteria [i.e., minimum inhibitory concentration (MIC) 1.25 mg/mL]. Acetone extract (unsaturated fatty acid-enriched) and tannin-like (dicarboxylic acid-enriched) substances inhibit both bacterial planktonic growth [MIC 1 and 3 mg/mL, respectively] and biofilm formation. The yarn lost its antibacterial activity once its surface lignin reached 20.1%, based on X-ray photoelectron spectroscopy. The proportion of fiber bundles at the fabricated yarn correlates positively with its surface lignin. Overall, this study paves the way to the use of bark-derived fiber bundles as a natural-based material for active (antibacterial and antibiofilm) wound dressings, upgrading this underappreciated bark residue from an energy source into high-value pharmaceutical use.

## Introduction

Chronic wounds causing acute problems for both patients and the healthcare system are estimated to account for approximately 1–3% of the total healthcare expenditure in developed countries.^1^ In future years, chronic wound-related burden is expected to further grow due to an aging population and sharply increasing occurrence of lifestyle diseases, such as obesity and diabetes, which contribute to wound healing delay and chronicization.^2^

Bacteria are an integral part of the human skin and their presence and replication in the wound typically does not prevent the healing process. However, the normal immune function is impaired in chronic wounds, shifting the balance in favor of bacteria and resulting in invasive wound infections.^3^ The prevalent form of chronic wound bacterial colonization is a biofilm—a complex structure that is formed by bacterial cells (10–20%) and their self-secreted extracellular matrix (80–90%).^4^ Biofilms are strongly associated with the failure of acute wound treatment and development of chronic wounds. They are extremely hard to be removed and can continuously interact with host immune cells and induce cytokine production, thus contributing to inflammatory processes and preventing healing.^5^ The current standard of care relies on wound debridement and antibacterial treatments including both topical and systemic antibiotics.^6^ The intrinsic resistance of bacterial biofilms and the increased prevalence of multidrug-resistant bacteria complicate infection treatment; thus, the development of alternative therapies is urgently needed. Antimicrobial-loaded dressings such as those containing silver, povidone iodine, or chlorhexidine are widely used in clinic and available from pharmacies. These materials are effective against a wide range of microorganisms including bacteria, fungi, protozoa, and viruses. However, in contrast to conventional antibiotics, their mechanism of action is not target-specific, and it may result in significant cytotoxicity to skin cells during the course of treatment including fibroblasts and keratinocytes.^7^

A growing field in the search for alternative solutions is natural product-based materials, which have been extensively studied. Many reported materials require a complex fabrication process, e.g., synthesis of polypeptides or production and incorporation of plant extracts, albeit others possess intrinsic antibacterial properties due to active substances, e.g., antibacterial peptides, honey, and propolis (Table 1). Several honey-containing products are commercially available, and more alternatives would be welcomed by clinicians and therefore important to develop. Energy willow grows effectively in abandoned peatlands that are cultivated, which are considered the best option for peatland rehabilitation.^8^ The bark represents 10–20% of the entire volume of the trees depending on the hybrid, age, and season,^9^ and for decades, it has mostly been utilized as energy supply, whereas another potential pharmaceutical application remained largely undiscovered. To date, the main wood-derived material suggested for application as wound dressings is based on nanocellulose.^10,11^ Although nanocellulose is inexpensive, biodegradable, and has remarkable biocompatibility properties, it lacks intrinsic antibacterial activity,^12^ requiring incorporation of additional bioactive components.

**Table 1:** Representative Examples of Antibiotic-Free Antibacterial Wound Dressings Using Nature-Derived and Nature-Inspired Substances

	material and active component	main properties	reference
synthesis of polypeptides	hydroxyethyl cellulose hydrogel loaded with thrombin-derived peptides	antibacterial activity (*S. aureus* and *E. coli*) and reduced inflammation	(13)
	hydrogel developed based on salep/poly(vinyl alcohol)	antibacterial activity (*S. aureus* and *E. coli*) and self-healing	(14)
	carboxyl-modified cellulosic hydrogel with covalently bound ε-poly-l-lysine	antibacterial activity (*S. aureus* and *E. coli*) and high biocompatibility with model mammalian cells	(15)
incorporation of bee products	polyurethane–hyaluronic acid nanofibrous wound dressing enriched with three different concentrations of ethanolic extract of propolis	antibacterial activity (*S. aureus* and *E. coli*), biocompatibility (fibroblast cells), and accelerated wound healing	(16)
	honey-loaded alginate/polyvinyl alcohol electrospun nanofibrous membranes	antibacterial activity (*S. aureus* and *E. coli*), antioxidant activity, and biocompatibility with model mammalian cells	(17)
incorporation of plant extracts	biopolymer films containing chitosan, eggshell membranes, soluble eggshell membranes, and extracts from *Thymus vulgaris* and *Origanum valgare*	antibacterial activity (*E. coli*), antioxidant activity, biodegradable, fluid absorption, and pH properties favorable for wound healing	(18)
	hydrogel films loaded with chlorogenic acid	antibacterial activity (*E. coli* and *S. aureus*) and wound healing performance evaluated by a mouse full-thickness wound model	(19)
	membrane hydrogels based on Kraft- and ionic-liquid-isolated lignins	antibacterial activity (*E. coli*) and antioxidant efficiency favorable for wound dressing materials	(20)
willow bark fiber bundles	inherent bioactive extractives (unsaturated fatty acid-rich), tannin-like (organic acid-rich), and lignin	antibacterial and antibiofilm activities against *S. aureus*	present study

Traditional folklore claims that willow bark provides a major defense against surrounding pathogens.^21^ Indeed, different wood macromolecular components have been reported to have antibacterial activity (Table 2). Lignin, a major component from the wood is active against multiple food-borne and human pathogenic microorganisms including *S. aureus*,^22,23^ acting presumably by damaging the cell membrane.^24^ Antimicrobial and antioxidant activity of lignins may vary depending on botanical species and specific fractions.^24^ Antibacterial activity of tannins isolated from different plants is also well documented.^25,26^ They have been shown to be active also against methicillin-resistant *S. aureus* strains.^27^ Tannins were reported to act by multiple mechanisms, including interference with bacterial metabolism and cell adhesion (reviewed in ref (28)) and were suggested as a promising component of biomaterials. Hemicelluloses, particularly the purified arabinoxylan fractions^29^ from *Plantago ovata* seed husk, have been shown to be effective against Gram-positive bacteria.^29^ Pectin is another known antibacterial agent that partially inhibits the growth of *S. aureus* and *P. aeruginosa*(30) due to its galacturonic acid residues.

**Table 2:** Summary of the Reported Bioactive Components from the Lignocellulosic Biomass against Pathogenic Bacterial Species

active component from wood	tested pathogen species	potential applications	origin/active component	reference
lignin	clinically isolated biofilm-forming bacteria *P. aeruginosa*, *S. aureus*, and *Serratia* sp. and laboratory strains *S. aureus*, *L. monocytogenes*, and *S. typhimurium*	a component of composite hydrogel to facilitate chronic wound healing	dehydrogenative polymer of coniferyl alcohol	(31)
	*E. coli*, *S. aureus*, *P. mirabilis*, *P. vulgaris*, *P. aeruginosa*, *E. aerogenes*, *B. thuringiensis*, and *S. mutans*	antimicrobial additive or agent in food, textile, or chemical industry	spruce and eucalyptus kraft and organosolv lignin	22
hemicelluloses	*E. coli*, *S. aureus*, and *P. aeruginosa*	antibacterial film wound dressings	arabinoxylan of *Plantago ovata* seed husk	29
	Gram-positive and Gram-negative species relevant for food industry	food and medical applications	almond gum hemicelluloses	(32)
pectin	*S. aureus*, *H. pylori*, *E. coli*, and *P*. *aeruginosa*	tissue and bone engineering	galacturonic acid residues of pectin from lemon peels	30
extracts	*E. coli*, *P. aeruginosa*, and *B. subtilis*	tissue engineering and wound dressing	giloy extract	(33)
tannins	*S. aureus* and *K. pneumoniae*	healing-promoting wound dressings	immature fruits of *Terminalia chebula*	(34)

In our previous work, we reported significant activity of the willow bark fiber bundles (WBFBs) against *S. aureus*,^35,36^ the predominant Gram-positive bacterial species found in infected wounds.^37^ Within this study, we investigated the potential use of wood bark-derived WBFBs as a renewable and sustainable alternative natural product-based wound dressing material with intrinsic antibacterial activity. Considering the importance of biofilm lifestyle in chronic wound formation, we specifically focused on the antibiofilm properties of WBFBs. Furthermore, we performed an in-depth chemical characterization and identification studies on key chemical contributors to the observed bioactivity.

## Materials and Methods

### Raw Materials and Chemicals

Two-year-old willow Klara hybrid stems were harvested from Carbons Finland Oy (Kouvola, Finland) on May 5, 2019. The bark was manually peeled and stored at −20 °C for further use. Acetone, arabinose, *N*,*O*-bis(trimethylsilyl)trifluoroacetamide (BSTFA) containing 10% trimethylchlorosilane (TMCS), chloroform, citric acid, dichloromethane, dioxane, dimethyl sulfoxide (DMSO), DMSO-*d*6, ethylenediaminetetraacetic acid (EDTA), ethanol, fructose, galactose, glucose, glyceryl trioleate, hydrochloric acid, hydrogen peroxide, mannose, methanol, pepsin, peracetic acid, pyridine-*d*5, rhamnose, sodium hydroxide, sodium methoxide, sodium sulphate, tetracosane (C24), and xylose were supplied from Sigma-Aldrich, Finland.

### Fractionation of the Willow Bark

#### Fractionation Scheme

**Figure 1:**
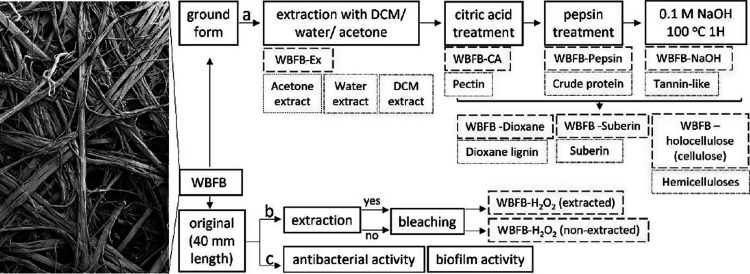
WBFB sample preparation for biological activity assessment. (a) Step-by-step fractionation process (solid black lines). Separating WBFBs into fractionated components (dotted lines) used for antibacterial activity assessment, along with the remaining solid residues and fiber bundles (dashed lines). WBFB-Ex refers to the solid residues after the sequential extraction with dichloromethane (DCM)/water/acetone. WBFB-CA refers to the solid residues after solvent extraction and citric acid (CA) extraction (complete removal of pectin and extractives). WBFB-Pepsin refers to the further removal of proteins. WBFB-NaOH refers to further removal of tannin and tannin-like substances. All these steps are considered as pretreatment for further sample preparation of dioxane lignin, suberin, and hemicelluloses. WBFB-Dioxane, WBFB-Suberin, and WBFB-holocellulose (cellulose), respectively, represent their associated solid residues. (b) Assessing the effects of extraction on antibacterial activity. (c) Assessing antibacterial activity of the raw material. For photographs of the fractionated components see Figure S1.

Well-aligned WBFBs were recovered using sodium bicarbonate at 100 °C as previously described.^35^ A part of the WBFB was applied for the bioactivity studies without any processing (Figure 1b), while another part of dry WBFBs was ground (1 mm mesh size) (Wiley Mill, USA) and individual fractions of water, acetone and dichloromethane extracts, pectin, tannin-like substances (tannin-like), dioxane lignin, suberin, and hemicelluloses were prepared (Figure 1a).

#### Extraction

WBFBs were successively extracted with dichloromethane, acetone, and water using a Soxhlet apparatus according to SCAN-CM 49:03 (2003). The organic solvents were removed from the extracts by evaporation in a fume hood overnight after which the samples were freeze-dried and preserved in a desiccator.

#### Pectin Removal and Recovery

Extracted WBFBs were treated with 1 wt % CA (pH 3) in an Erlenmeyer flask at 80 °C for 1 h. The reaction was stopped by cooling the flask in an ice bath and the reaction mixture was filtered on a Büchner funnel. The solid residue (WBFB-CA) was washed successively with water (until neutral pH) and acetone for a complete removal of citric acid and then freeze-dried. The pectin-rich filtrate was mixed with absolute ethanol (1:3 volume ratio) and kept at 4 °C for pectin precipitation. The freeze-dried crude pectin was further dialyzed (Biosharp, 6–8 kDa molecular weight cutoff) for 96 h to eliminate any small molecules. The dialyzed pectin was lyophilized and preserved in a desiccator.

#### Removal of Crude Protein and Tannin-like Substances

WBFB-CA was treated with 1% pepsin in 0.1 M HCl (liquid-to-solid ratio 25:1) at 37 °C for 16 h. The reaction mixture was filtered on a Büchner funnel. The filtrate was lyophilized, and the solid residue (WBFB-Pepsin) was washed with hot water until the washing liquid became neutral. WBFB-Pepsin was further treated with 0.1 M NaOH under nitrogen flow at 100 °C for 1 h with the target of removing tannin-like without deacetylating the hemicelluloses. The solid residue (WBFB-NaOH) was separated by filtration and washed with water until pH 6 was achieved for the filtrate.

#### Dioxane Lignin and Suberin Recovery

The alkali-extracted bark was prepared for dioxane lignin purification.^38^ Specifically, WBFB-NaOH (l:s, 30:1) was submitted to three times sequential extractions (30 min each) under a dioxane–water (9:1, v/v) mixture containing 0.1 M HCl under the reflux condensing system in a nitrogen atmosphere at 90–95 °C for a period of 30 min. Crucibles (pore size 3–4) were employed to collect the purified fractions. The fourth extraction was conducted under the dioxane/water mixture alone. Each portion of the dioxane–water extract was concentrated using a rotavapor separately (to around 40 mL) and then all the dioxane lignin-soluble concentrates were combined and precipitated under cold water. Dioxane lignin was then centrifuged (8000 rpm) and freeze-dried before storing in a desiccator for further characterization. Suberin recovery was conducted using alkaline methanolysis with sodium methoxide, as described in detail in the literature.^39,40^

#### Peracetic Acid Delignification and Recovery of Hemicelluloses and Cellulose

Approximately 5.5 g of WBFB-NaOH (l:s, 30:1) was placed inside a plastic bag containing 10% peracetic acid (pH 4) under 85 °C for 1 h. The reaction was quenched using ice and the solid product (i.e., holocellulose) was filtered through a Büchner funnel using a solvent of 10% acetic acid, water, and acetone, respectively. Then, the lyophilized holocellulose was extracted with DMSO (l:s, 20:1) at 50 °C for 12 h. Following the first DMSO extraction, the solid was vacuumed and washed with approximately 300 mL of ethanol/methanol mixture (v/v, 7/3). Then, the solid was transferred back to the same Erlenmeyer flask again for the second round of DMSO extraction. These filtrates were combined at a relative ratio of the solvents 3:5 (v:v, ethanol–methanol mixture:DMSO). The final precipitation was then conducted by adjusting the pH to 3. The hemicelluloses were precipitated in a fridge (+4 °C). The ethanol–methanol mixture was added as a countersolvent to lower the solubility of hemicelluloses. Furthermore, the acidic pH results in protonation of the carboxylic acid group of xylan for facilitating its precipitation. The final centrifugation was performed using the solvent methanol with the aim of collecting the solid matter and removing DMSO to replace it with an easier to remove solvent (i.e., ethanol–methanol mixture). The lyophilized hemicelluloses were preserved under a desiccator for further bioactivity characterization.

#### Bleaching with H_2_O_2_

Both the extracted and nonextracted WBFBs (Figure 1) were bleached (l:s, 1:50) by treatment with 35% hydrogen peroxide (3 mL/L) and a sequestering agent (EDTA 1 g/L) in a water bath (pH 11) at a temperature of 85 °C for 1 h. Then, washing and freeze-drying were performed afterward to recapture WBFB-H_2_O_2_ (extracted) and WBFB-H_2_O_2_ (nonextracted) (Figure S1) for further characterization.^41^ Bleaching is performed to assess the contribution of chromophores (or natural colorants) into the shown bioactivity of the WBFB.

#### Manufacturing of the Yarn Samples

Blended yarns were manufactured following the blend weight ratio of WBFB and lyocell: 50 WBFB/50 lyocell, 30 WBFB/70 lyocell, and 10 WBFB/90 lyocell. First, WBFBs of 40 mm staple length were separated manually into individual fibers or thinner bundles. Then, 20 g fiber batches from lyocell (Tencel 1.3 dtex, 38 mm, Lenzing AG, Austria) and WBFBs (blend ratios 90/10, 70/30, and 50/50, respectively) were laid out in layers (i.e., 2 g of WBFBs on top of 18 g of lyocell, 10 WBFB/90 lyocell) to the conveyor belt of a carding machine (Carding Machine 337A, MESDAN Lab, MESDAN S.p.A., Italy) and 10 WBFB/90 lyocell were carded to obtain a thin fiber web. The carding phase mixed and homogenized fiber types together. The fiber web was formed into a sliver that was further elongated using a draw frame (Stiro Roving Lab 3371, MESDAN Lab, MESDAN S.p.A, Italy). The elongated sliver was folded twice and elongated again into a thinner sliver and twice-folded again to ensure as homogenous a sliver as possible. Finally, the twice-folded sliver was elongated and formed into a false-twisted roving (preyarn). The 100% lyocell roving was prepared similarly as blended yarns but without blending with WBFBs. For reference purposes, bleached cotton yarn was used as received from Orneule Oy (Finland). For antimicrobial testing, 100 WBFBs were tied together with a negligible amount of yarn.

### Biological Testing

#### Bacterial Strains

Human wound-isolated *S. aureus* DSM 25691 (methicillin-resistant) and DSM 28763 strains were purchased from the DSMZ German Collection of Microorganisms and Cell Cultures GmbH (Germany). Fresh cultures were initiated from −80 °C glycerol stocks on Mueller Hinton agar (MHA, BD) plates monthly, from which fresh weekly cultures were prepared each week.

#### Antibacterial Activity of WBFBs

The assay was performed following the absorption method of the European Standard EN ISO 20743:2013 in *S. aureus* DSM 28763 and DSM 25691, and 0.4 g of cotton yarn (control) and test materials were weighed and sterilized in an autoclave prior to the experiment.

Overnight liquid culture was prepared by transferring one colony from the freshly grown MHA plate into 20 mL of tryptone soy broth (TSB) in 50 mL of conical centrifuge tube with a filter cap for aeration. The liquid culture was incubated for 18–24 h at 37 °C with a shaking rate of 200 rpm. On the day of the experiment, this overnight culture was diluted 1:100 (50 μL in 5 mL) in fresh TSB and incubated for 2 h at 37 °C with shaking at 200 rpm. After the incubation, the bacterial concentration was determined by measuring the absorbance at 620 nm with a Multiskan GO (Thermo Fischer Scientific) plate reader. Bacterial concentration was adjusted to 2 × 10^5^ CFU/mL with fresh TSB.

Samples were placed into 50 mL conical centrifuge tubes, prewetted with 500 μL of sterile MilliQ water, and inoculated with 200 μL of bacterial suspension at different spots of the yarn bundle. Each sample was prepared in duplicate. The first set of samples was analyzed immediately after inoculation. The second set of samples was incubated for 24 h at 37 °C. For the analysis, 20 mL of saline solution (0.9%) was added into the vials, and the vials were mixed 5 × 5 s with vortexing. Tenfold dilution series (500 μL in 5 mL) were prepared in TSB, and a range of dilutions were plated by mixing 1 mL of dilution with 15 mL of melted tryptone soy agar (TSA) cooled to 45 °C. Each dilution was plated in duplicate. Plates were incubated at 37 °C for 24 h. Colonies were counted from plates containing 30–300 bacterial colonies. The experiment was repeated at least three times for each strain. Antibacterial activity (*A*) was calculated using the formula:
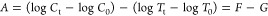
where *F* = (log *C*_t_ – log *C*_0_) is the growth rate on the cotton control; log *C*_t_ is the average of common logarithm of the number of bacteria obtained from the cotton specimen after 24 h incubation; log *C*_0_ is the average of common logarithm of the number of bacteria obtained from the cotton specimen immediately after inoculation; *G* = (log *T*_t_ – log *T*_0_) is the growth rate of the test samples; log *T*_t_ is the average of common logarithm of the number of bacteria obtained from the test specimen after 24 h incubation; and log *T*_0_ is the average of common logarithm of the number of bacteria obtained from the test specimen immediately after inoculation.

#### Antibacterial Activity of the Individual Components Recovered from WBFBs

Compounds were tested in both *S. aureus* strains using broth microdilution assay recommendations outlined by the CLSI standards.^42^ First, we performed a screening experiment at a concentration of 1 and/or 0.5 or 2 mg/mL. The primary screening concentrations were dictated by solubility and sample availability. Inhibition percentage (%) was calculated relative to maximum growth diluent-treated control. For compounds demonstrating over 90% bacterial growth inhibition, dose–response experiments were performed. A range of compound concentrations (twofold dilutions) was prepared and tested following the same procedure. Dose–response experiments were performed 2–3 times. All experiments were performed in triplicate wells. The detailed procedure is described in the Supporting Information.

#### Characterization of Wound-Isolated *S. aureus* Strains for Biofilm Formation

Bacterial suspension for biofilm formation experiments with a concentration of 1 × 10^6^ CFU/mL was prepared as described in section titled “Antibacterial Activity of the Individual Components Recovered from WBFB” in the Supporting Information, and added to a 96-well plate, 200 μL per well. In all biofilm experiments, we used TSB supplemented with 1% glucose, as the defined medium for optimal biofilm growth of *S. aureus*.^43^ The plates were then incubated for 24 h at 37 °C. The formed biofilm was evaluated using three approaches: colony (CFU) counting, biomass assessment by crystal violet (CV) assay, and cell metabolic activity assessment by resazurin assay. We used standard procedures,^43^ described in detail in the Supporting Information. Four independent experiments with six replicate wells were performed for CV and resazurin assays. CFU counting results are from three independent experiments with four replicate wells.

#### Antibiofilm Activity of WBFBs and Selected Compounds

Antibiofilm assays were performed with the strongest biofilm former strain, *S. aureus* DSM 28763 (Figure S2). Willow bark samples (100 WBFB, 30/70 WBFB/lyocell, and 50/50 WBFB/lyocell) were weighed, manually shaped into small round bundles, and autoclaved prior to the experiments. The weight of each sample was 30 ± 1 mg and placed into the well of a sterile 96-well plate containing the bacterial suspension prepared as described above. After incubation for 24 h at 37 °C with a shaking speed of 250 rpm (PST-60HL-4 Thermo-Shaker), samples and media were removed from the wells and the biofilm was washed with 1× PBS. Treated and nontreated biofilms were quantified using CV and CFU assays as described in detail in the Supporting Information. Three independent experiments were performed with six technical replicates.

Individually isolated compounds (acetone extract, tannin-like substances, and dioxane lignin) from WBFBs were assessed for antibiofilm properties at final concentrations of 1, 1.25, and 3 mg/mL (i.e., minimum inhibitory concentration (MIC) values), respectively. Acetone extract and dioxane lignin stocks were prepared by diluting samples in DMSO to 50 mg/mL, while tannin-like substances were diluted in sterile water to 25 mg/mL. An equal volume of the bacterial inoculum (2 × 10^6^ CFU/mL) and diluted sample were added in each well of sterile 96-well microplates. Three independent experiments were performed with six replicate wells for CV and resazurin assays and three replicates for the CFU assay. Maximum biofilm formation was determined from untreated control wells. These wells contained media proportionally supplemented with the diluent used for compound stock preparation. Wells with media only were used as the background control.

### Analytical Techniques

#### Nuclear Magnetic Resonance Spectroscopy

1D ^1^H and ^13^C and 2D ^1^H–^13^C heteronuclear single quantum coherence (HSQC) spectra were acquired using a 400 MHz Bruker Avance III spectrometer. Acetone-*d*6 (δC 29.92, δH 2.05 ppm) and DMSO-*d*6/pyridine-*d*5^44^ (δC 39.5, δH 2.49 ppm) were used as a calibration solvent for acetone extract and dioxane lignin (Figure 1), respectively. Pyridine-*d*5 has been reported to improve the intensities and resolution of the NMR spectrum compared to DMSO-*d*6 alone due to the enhanced expansion of the cell wall.^44^ HSQC spectra were acquired using 2 s *d*1, 1 K data points, 256 t1 increments, and 100 transients, an adiabatic version was adopted (hsqcetgpsisp.2 pulse sequence from the Bruker Library). ^1^H nuclear magnetic resonance (NMR) spectroscopy was acquired using a spectrum width of 16 ppm, *d*1 of 1 s and 32 K data points. The following parameters were used for ^13^C: a spectral width of 236 ppm, *d*1 of 2 s, and 65 K transients of 64 K data points. The spectral images were processed using TopSpin 4.0.

#### Chemical Composition

The chemical composition of the solid residue (Figure 1) was analyzed according to NREL/TP-510-42618. The quantitation of the hydrolyzed monosaccharide was determined using the high-performance anion-exchange chromatography with pulsed amperometric detection (HPAEC-PAD). The detailed equipment parameters used are described in our previous work. Determination of galacturonic acid by acid hydrolysis can bring unwanted degradation of galacturonic acid;^45^ thus, the recovery factor of galacturonic acid (i.e., 59.2 ± 0.007%)^36^ was taken into calculation.

#### X-ray Photoelectron Spectroscopy

X-ray photoelectron spectroscopy (XPS, Kratos Axis Supra instrument) was employed to assess the atomic surface’s compositional profile of the captured WBFBs and their derived fabrics. Pure “Whatman” cellulose paper was used as a reference. The atomic concentrations were calculated by CasaXPS software, and the energy shift correction was calibrated with reference to the C–C peak (284.8 eV). The Shirley background and Voigt function have been used to interpret results according to the literature.^46^

#### Gas Chromatography–Mass Spectrometry

Approximately 10 mg of isolated acetone extract and tannin-like were dissolved in 0.5 mL of pyridine containing 1 mg/mL tetracosane (C24) as the internal standard; 0.3 mL of BSTFA containing 10% TMCS was then introduced into the mixture for silylation. The specific temperature program of gas chromatography–mass spectrometry (GC–MS) was described previously where the column HP-5MS (30 m × 0.25 mm, i.d. 0.25 μm) was employed.^40^ Their mass fragments were referenced with the NIST Chemistry WebBook.

### Result Analysis Methodology

“Mean” and “standard deviation” have been applied to calculate the average of chemical composition of the components through HPAEC-PAD; mass balance; and quantification of the acetone extract through GC–MS based on triplicate-independent measurements per sample, which only shows the repeatability (or variance) of the results. The coefficient of variance (i.e., standard deviation/mean) is small for all cases with measurable contents. The largest coefficient of variance for any nonzero content is less than 5%.

In biological assays, data plots and statistical analysis were done using OriginPro Graphing and Analysis software, version 2021b (OriginPro). Normal distribution was tested using the Shapiro–Wilk test. Grubb’s test was used to detect outliers. Biofilm formation data were normally distributed, and the analysis of variance (ANOVA) test was performed to identify differences between the two wound *S. aureus* strains. For antibiofilm results, mean values of biomass were not normally distributed for all groups; thus, the Kruskal–Wallis nonparametric test was used for analyzing differences between groups. CFU were converted into log_10_ and analyzed for differences using ANOVA with the post-hoc Bonferroni test for the multiple mean comparisons. Significant differences were assigned when *p* < 0.05.

## Results and Discussion

### Bioactivity Evaluation of WBFBs

#### Antibacterial Activity of WBFBs

According to our earlier studies WBFBs showed strong antibacterial activity against laboratory strain *S. aureus* ATCC 29213 typically used in antibacterial screenings.^35^ As susceptibility to antibacterial compounds may vary considerably between strains, we first verified the antibacterial activity of WBFBs in two clinical strains isolated from infected wound *S. aureus* DSM 28763 and DSM 25691. The former strain is characterized by the ability to form biofilms, while the latter strain is resistant to methicillin and other antibiotics. We then mixed WBFBs with lyocell and determined the minimum WBFB content that is required for retaining antibacterial properties. Lyocell is a wood-derived, environmentally friendly, and recyclable material, which has been reported to have favorable wound dressing properties. In particular, it has been shown that the growth of *S. aureus* on nonwoven lyocell materials is reduced, presumably due to high moisture absorbance.^47^ Our experiments demonstrated no antibacterial properties of pure lyocell materials, probably due to experimental procedures which include a prewetting step. Both 50/50 WBFB/lyocell and 30/70 WBFB/lyocell demonstrated exceptionally strong antibacterial activity in tested strains as no viable bacteria were detected in these samples after 24 h incubation (Table 3). When the WBFB content was reduced to 10% (i.e., sample 10/90 WBFB/lyocell), antibacterial activity dropped from strong to significant in case of *S. aureus* DSM 25691 and was completely abolished for the DSM 28763 strain. Therefore, the material was proven to eradicate clinical *S. aureus* strains at a minimum of 30% WBFB content. Prewetting of the material did not affect antibacterial properties, suggesting that such a material can retain antibacterial properties also in wet wounds.

**Table 3:** Surface Lignin Coverage (or C–C %, Evaluated by XPS) and Antibacterial Activities against Two Wound-Isolated *S. aureus* Strains of WBFBs, Bleached Yarns (WBFB-H_2_O_2_), and Blend WBFB/Lyocell Yarns in Comparison to Lignin-Free WBFB-Cellulose and Pure Lyocell^a^

	surface lignin coverage		*S. aureus* DSM 28763		*S. aureus* DSM 25691	
sample	C–C % ± SD	surface lignin (%)	antibacterial value *A*	antibacterial effect	antibacterial value *A*	antibacterial effect
WBFB	23.0 ± 0.9	42.5	ND	strong^b^	ND	strong^b^
WBFB-H_2_O_2_ (nonextracted)	18.0 ± 0.3	32.5	8.6 (9.3, 7.9, 8.5)	strong	8.5 (9.0, 6.2, 10.4)	strong
WBFB-H_2_O_2_ (extracted)	23.9 ± 0.1	44.4	4.5 (7.3, 3.9, 2.2)	significant to strong	4.6 (5.3, 2.0, 6.3)	significant to strong
50/50 WBFB/lyocell	12.7 ± 0.6	21.9	8.0 (7.9, 8.1)	strong	8.6 (9.3, 8.0)	strong
30/70 WBFB/lyocell	12.1 ± 1.3	20.7	8.0 (7.9, 8.1)	strong	8.6 (9.3, 7.9)	strong
10/90 WBFB/lyocell	11.7 ± 0.1	20.1	0.4 (0.6, 0.2)	no effect	2.6 (2.8, 2.5)	significant
WBFB-cellulose	6.0 ± 0.1	8.5	1.9 (2.0, 1.8)	no effect		
100 lyocell	9.1 ± 0.3	14.7	0.3 (0.1, 0.5)	no effect	0.6 (1.0, 0.1)	no effect
Whatman cellulose (reference)	3.5 ± 0.1	3.5	ND	ND	ND	ND

a The table shows mean antibacterial activity value (*A*) as well as values obtained in 2–3 independent experiments (in brackets). For detailed chemical composition of the analyzed fiber materials, see Figure S3. No inhibition: (*A* < 2); significant inhibition: (2 ≤ *A* < 3); strong inhibition: (*A* ≥ 3). Results are relative to cotton (negative control). SD and ND are abbreviations for “standard deviation” and “not determined” respectively. For other abbreviations see Figure 1.

b Although antibacterial activity of the pure WBFB was not directly assessed here, it was demonstrated earlier in other *S. aureus* strains.^35^ Based on the 50/50 WBFB/lyocell sample data, we can suggest strong antibacterial effect of pure WBFBs in clinical strains used in this study.

We previously observed growth inhibition of several bacterial species, including *S. aureus*, on willow bark samples, e.g., for water extracts from the bark of 16 *Salix* clones^48^ and methanolic extracts from stem bark and leaves of *Salix alba*.^49^ These effects have been mainly attributed to salicinoids and various polyphenols. However, these compounds were depleted from WBFBs during their manufacturing process, and nevertheless, they retained strong antibacterial activity. To elucidate the role of individual WBFB components in the antibacterial activity of the material, we studied antibacterial activities of lignin-free (i.e., WBFB-cellulose) and bleached samples (WBFB-H_2_O_2_ extracted and nonextracted) (Figure 1). WBFB-cellulose lost its antibacterial activity (Table 3). WBFB-H_2_O_2_ (extracted) (color light yellow) retained roughly half of the nonextracted sample activity (color dark brown), implying a key role of acetone extracts and possibly chromophores. The decrease in the antibacterial activity was significant, albeit not as strong as that noted for WBFB-cellulose when lignin and tannin-like were removed in addition to extractives (Figure 1).

XPS has been previously adopted to show the atomic surface (top 10 nm) chemical profile of the WBFB.^50^ The processed fraction of the binding energy component C–C (284.8 eV) was used as a quantitative marker of the surface lignin content. The nominal lignin surface coverage was calculated from the noncellulosic component (i.e., C–C% of the real sample and our reference Whatman cellulose) as previously described.^51−53^ As expected, the content of WBFBs (from 10 to 30 wt % and progressively to 50 wt %) is proportional to the estimated surface lignin content (Table 5). When the acetone extraction was performed before the bleaching sequences, WBFB-H_2_O_2_ (extracted) with a high surface lignin content of 44.4% was obtained. The lignin/condensed tannin might achieve a higher exposure at the surface, and this might contribute positively to the surface “lignin” content. Clearly, the antibacterial activity was completely lost once the estimated nominal surface lignin coverage (Table 5) was ca. 20.1% (observed in 10/90 WBFB/lyocell) in comparison to ca. 20.7% (observed in 30/70 WBFB/lyocell).

#### Antibiofilm Activity of WBFBs

Considering the critical role of biofilm lifestyle in chronic wounds, it is essential to assess the antibiofilm properties of WBFBs. Prior to these experiments, *S. aureus* strains DSM 28763 and DSM 25691 were characterized for their ability to form biofilms (Figure S2). Three methods commonly used for biofilm quantification were applied: CV for total biomass, resazurin for the evaluation of metabolic activity of biofilm-forming bacterial cells, and CFU assay for quantification of bacterial cells within a biofilm. *S. aureus* DSM 25691 was classified as a weak biofilm former while DSM 28763 as a moderate biofilm former according to the suggested criteria.^54^ The fact that an increased biofilm mass was produced by DSM 28753, but cell numbers were not proportionally higher, could imply that this strain probably has a higher production and/or aggregation of extracellular substances in the biofilm matrix, which could lead to a more challenging biofilm-related infection to treat. Therefore, the strain DSM 28763 was selected as a model for characterization of antibiofilm properties of WBFBs.

**Figure 2:**
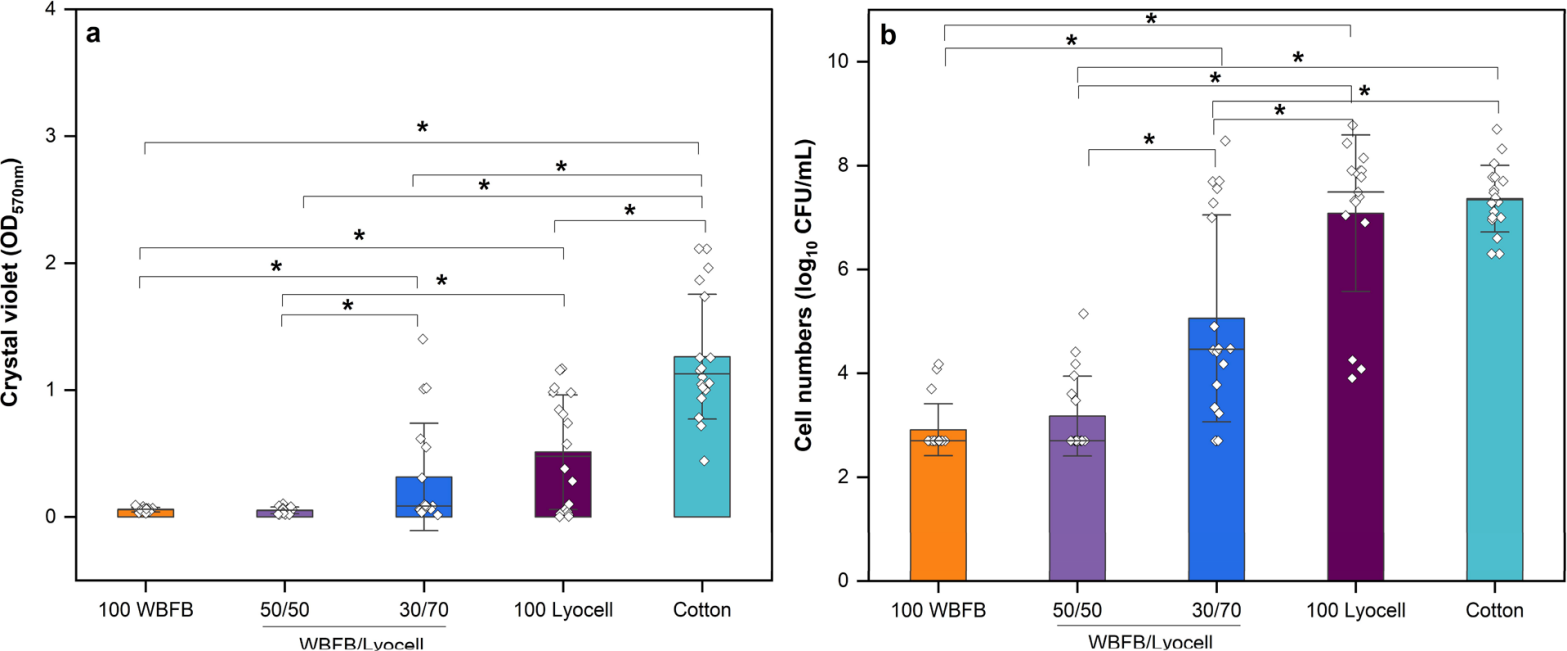
Inhibition of the *S. aureus* DSM 28763 biofilm formation by WBFBs. (a) Biofilm mass measured by CV assay after incubation with WBFB/lyocell yarns. (b) Log_10_ of biofilm cell numbers measured by CFU/mL after incubation with WBFB/lyocell yarns. The bars represent means of three independent biological experiments with six technical replicates for CV and three for CFU mean ± SD. Horizontal lines represent median. * denotes for statistical differences between samples (*p* < 0.05). For other abbreviations see Table 3.

The effect of pure WBFBs and WBFBs in combination with lyocell on total biomass and numbers of bacteria within the biofilm formed on polystyrene well plates in the presence of the material was evaluated. Figure 2a shows that incubation with 100 WBFB and 50/50 WBFB/lyocell resulted in 95.3 and 90.2% decrease in biofilm mass, respectively, in comparison to cotton control (*p* < 0.05). This reduction in biomass was also significantly higher than that produced by other sample blends (i.e., 30/70 WBFB/lyocell and 100 lyocell). Biofilm inhibition above 90% is considered of biological and medical significance, as highlighted in a recent review on combination therapies for biofilm-related infection.^55^ 30/70 WBFB/lyocell showed a moderate biomass reduction (i.e., 75%). It should be noted that 100 lyocell also showed some effect, reducing the biofilm mass by 59.5%, which was significantly different from cotton. This effect could be inherent to the methodology used (i.e., fibers touching the biofilm formed at the bottom of the well), which is illustrated by data set variability of individual replicates (Figure 2a). However, 100 lyocell had no effect on total bacterial cell numbers, which highlights the importance of using different assays to evaluate antibiofilm properties, similarly as demonstrated previously.^56^

In relation to bacterial cell numbers in the biofilm, a reduction of more than 4 log_10_ CFU/mL was achieved by 100 WBFB and 50/50 WBFB/lyocell, which accounts for a reduction in 99.99% of biofilm bacterial population. For a significant bactericidal effect, the agent under investigation must achieve ≥3 log_10_ CFU/mL reduction. A decrease of 20% on WBFB contents (i.e., 30/70 WBFB/Lyocell) had a significant decrease in the antibiofilm property when compared to 100 WBFB and 50/50 WBFB/lyocell. (Figure 2) Still, samples containing at least 30% of WBFBs significantly decreased the bacteria population in the biofilm when compared to lyocell or cotton (*p* < 0.05) (Figure 2b). Overall, yarns containing at least 50% of WBFBs would hinder biofilm formation of *S. aureus* on wounds. The effect on biofilm eradication was beyond the scope of this work.

Importantly, as the applied experimental procedure quantified the biofilm formed on the well plate, and not on the sample itself, it could only detect the effects of components diffusible in the water environment. Therefore, structural components of the material, such as lignin, do not contribute to the antibiofilm effect detected by this method. The observed activity of WBFBs provides evidence that the material can also affect bacterial biofilms that are not in direct contact with the wound dressing.

### Mass Balance and Bioactivities of the Purified Compounds

#### Mass Balance and Chemical Composition

Knowledge of a bioactive material’s chemistry is considered key for understanding its antibacterial performance. The activity of WBFBs could possibly be attributed to macromolecules like dioxane lignin, pectin, suberin, extractives, hemicelluloses, and tannin-like substances. Therefore, we deconstructed WBFBs and their full mass balance is presented in Table 4. In addition to the main wood components (i.e., cellulose, hemicelluloses, and dioxane lignin accounted for 58 wt %), pectin and extracts weighted for 1.9 and 0.7 wt %, respectively. Crude protein and suberin represents merely 0.4 and 0.1 wt %, respectively, indicating that the interactions between the lignin, carbohydrates (cellulose and hemicelluloses), pectin, and suberin is possibly present also here in WBFBs; although similar linkage interactions have been reported for the plant secondary cell walls of wood,^57,58^ the knowledge of their specific molecular-level interaction is out of scope of this study. Starch is tentatively identified as part of the “rest” at Table 4, which can be supported by the relatively high abundance of starch in the native form of willow bark by Raman spectroscopy.^59^

**Table 4:** Mass Balance of the WBFBs^a^

component	content %
cellulose	50.8 ± 0.1
hemicelluloses	5.2 ± 2.5
dioxane lignin	2.5 ± 0.4
pectin	1.9 ± 0.6
water extract	0.5 ± 0.11
crude protein	0.4 ± 0.03
DCM extract	0.1 ± 0.03
acetone extract	0.1 ± 0.01
suberin	0.1(ND)
sum of purified components	61.4
rest (e.g., tannin-like)	38.6

a “rest” refers to the unquantified tannin-like substances, ash, and other high molecular weight macromolecules (e.g., starch). The content % value represents the mean of three independent measurements ± SD. SD and ND abbreviations for “standard deviation” and “not determined”, respectively.

### Fractionation of WBFBs into Their Deconstructed Components

**Figure 3:**
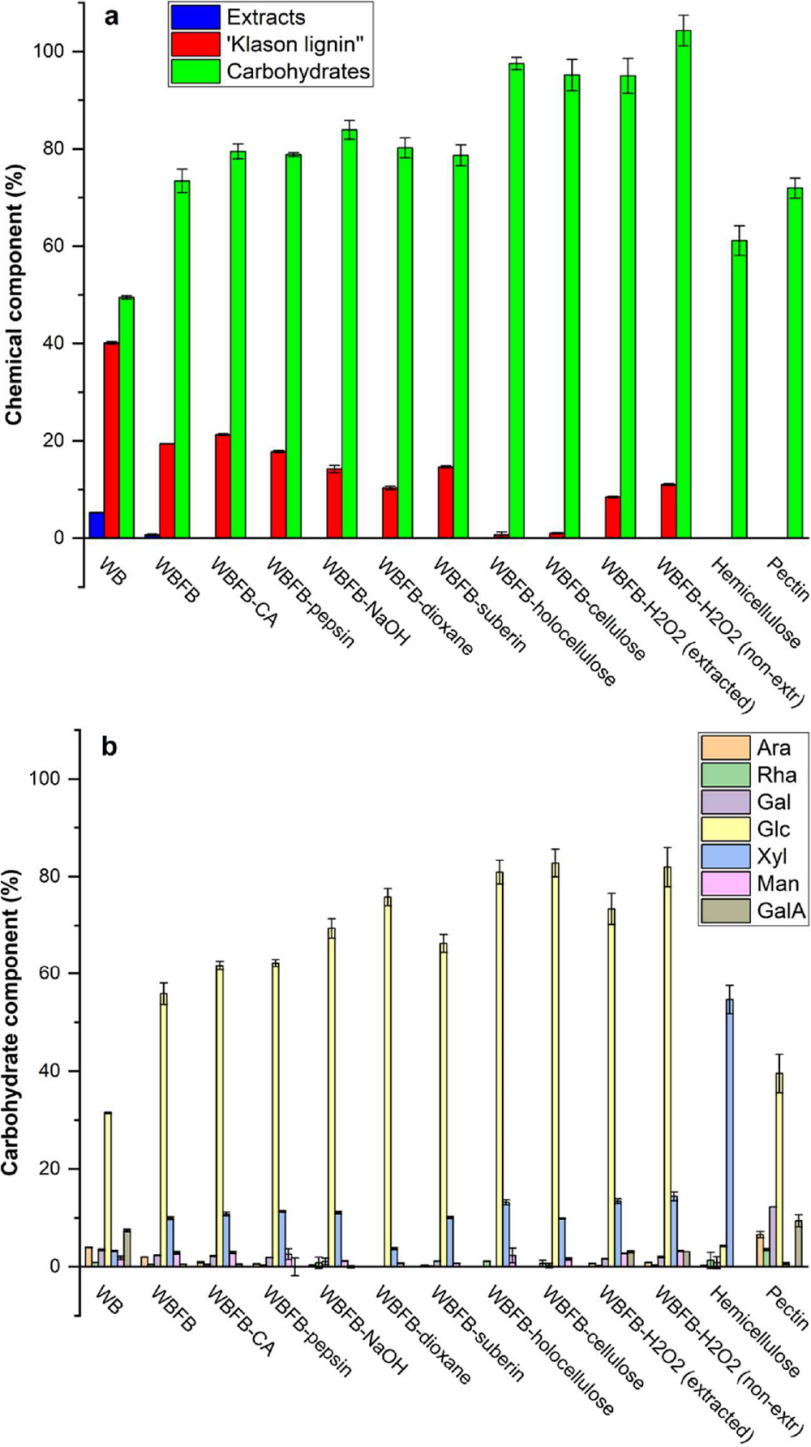
Chemical composition of the blended yarns in comparison with the WB, WBFBs, purified hemicelluloses, and pectin. (a) Overall chemical composition (% of the original dry mass). (b) Carbohydrate component (% of anhydro sugars in the original dry mass). Abbreviations: arabinose (Ara), rhamnose (Rha), galactose (Gal), glucose (Glc), xylose (Xyl), mannose (Man), galacturonic acid (GalA), and citric acid (CA). For other abbreviations see Figure 1.

The purified hemicellulose yield (5.2%, Table 4) contains xylose as its main monosaccharide (Figure 3). Lignin removal is significant particularly comparing the “Klason lignin” content between WBFB-NaOH (14%) and WBFB-holocellulose (1%). Additionally, the “Klason lignin” of WBFB-dioxane witnessed one third reduction in comparison to that of WBFB-NaOH, which indicates that the recovered lignin (2.5 wt %) might be a portion of the complete “Klason Lignin” or the presence of “Klason Lignin” at the WBFB maybe originated from some other condensed components like protein or starch.^60^ The characteristic sugars (i.e., galacturonic acid, arabinose, rhamnose, and galactose) of pectin were present in the recovered “pectin”, which proved the success of the applied CA extraction for binding calcium ions.^61^ The high abundance of glucose in the pectin fraction was possibly associated with the coextracted starch, which has previously been noted from the pectin fractions of willow bark.^36^ The almost nonpresence of galacturonic acid at WBFB-CA indicates the complete recovery of pectin with a yield of 1.9%. Overall, these results demonstrate the successful maximum preservation of the structural components from WBFBs, which lays out the key foundation for the follow-up bioactivity assessment of their deconstructed components.

#### Antibacterial and Antibiofilm Activities of Purified Components

The antibacterial activity of the individual WBFB components against *S. aureus* DSM 28763 was determined by the broth microdilution assay as described in the Supporting Information. Dioxane lignin, acetone extract, and tannin-like substances showed the highest antimicrobial activity among all the individual components (Table 5). The MIC required to achieve a 90% bacterial growth inhibition was determined for these components with acetone extract having the lowest value (1 mg/mL; Table 5). Although the antibacterial activity of lignin is well documented, it was shown to vary between botanical species and part of the plant,^23^ as well as to be dependent on the extraction method. For example, isolated lignin from grass failed to inhibit bacterial growth, but wood lignins (e.g., from bamboo, eucalyptus, beech, and spruce/pine isolates) showed antibacterial activity against several bacterial species including *S. aureus* (Table 2), corroborating with our data.

**Table 5:** Antibacterial Activity of Individual Components Isolated from WBFBs against *S. aureus* DSM 28763

	primary screening^a^		
sample	concentration (mg/mL)	antibacterial effect	MIC (mg/mL)
components recovered from WBFBs			
acetone extract	0.5	partial inhibition	1
	1	strong inhibition	
tannin-like substances	1	partial inhibition	3
DCM extract	0.5	no inhibition	
hemicelluloses	0.5	no inhibition	
	1	no inhibition	
dioxane lignin	0.5	partial inhibition	1.25
	1	partial inhibition	
pectin	0.5	no inhibition	
	1	no inhibition	
suberin	0.05^b^	no inhibition	
water extract	0.5	no inhibition	
authentic compounds (identified as a part of acetone extract)			
levoglucosan	1	no inhibition	
	2	no inhibition	
protocatechuic acid	1	no inhibition	
	2	partial inhibition	

a Primary screening was performed once at the indicated concentrations. For the compounds that showed visible inhibition of bacterial growth, MIC was determined in dose–response assays, and 2–3 independent dose–response experiments were performed.

b Indicates low solubility.

The components (Table 5) reported to be bioactive in the literature did not all show antibacterial activity in this study. Although antibacterial properties of hemicelluloses and pectin were reported by others (Table 2), they displayed no detectable activity in our study. The discrepancies in bacteriological assessments of nature-derived materials are common and may arise from multiple factors, including differences in chemistry of the molecules isolated from different species, their tested concentrations as well as differences in methodology, and used strains. For example, the disk diffusion assay or serial dilution followed by CFU counting is capable of detecting weaker (partial) antibacterial activity, whereas broth microdilution assays used in this study are designed to detect strong activity resulting in logarithmic-scale inhibition of bacterial growth. In addition, concentrations tested with this approach failed to exceed 2 mg/mL due to solvent and solubility limitations. Using a disk diffusion approach, hemicelluloses at the concentrations above 20 mg/mL have been shown to have moderate to strong antibacterial effects on multiple Gram-positive and Gram-negative bacterial species, including *S. aureus*.^32^ Therefore, considering the high presence of hemicelluloses and pectin in WBFBs (Table 4) and the less-destructive purification protocol, they might contribute to the overall antibacterial activity of WBFBs even though their antibacterial activity is relatively weak and was not detected by the approach used in this study. No shown bioactivity of suberin might be explained by the fact that the native form of suberin macromolecules have been decomposed into their monomers by the saponification process.^39,40^ In addition, it cannot be excluded that uncharacterized components could play a role alone or in combination with substances shown to be active in our study.

**Figure 4:**
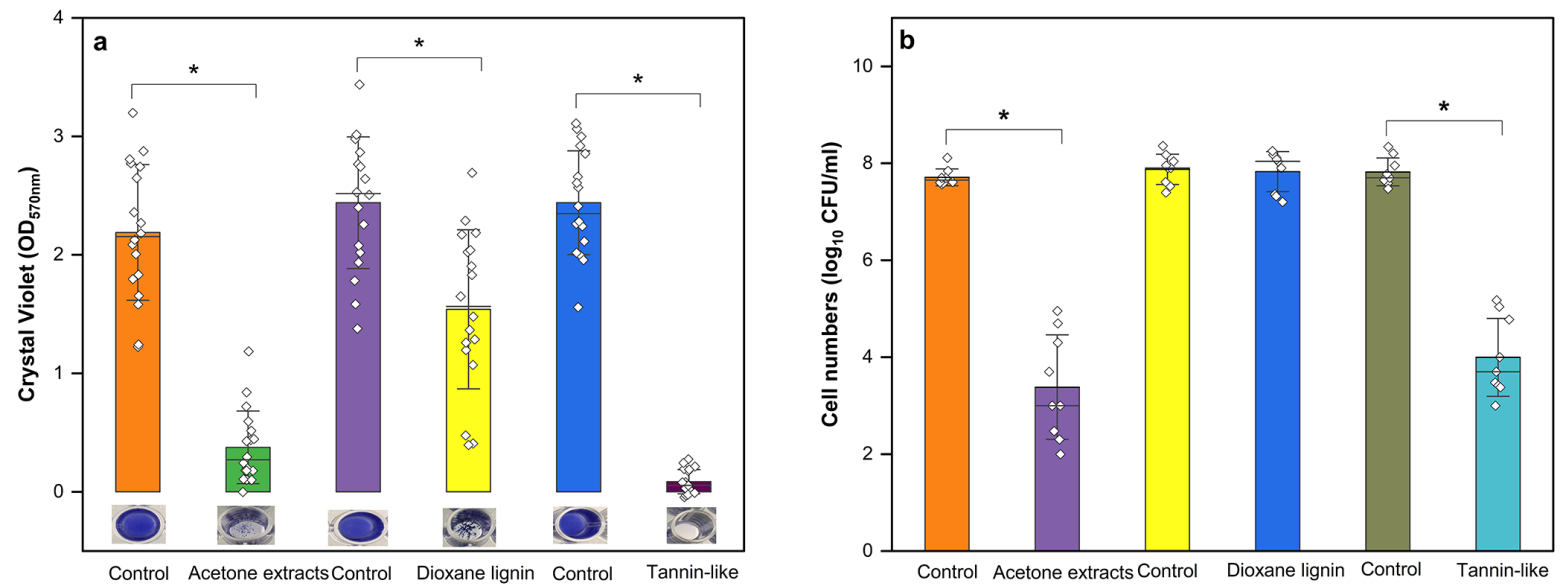
Inhibition of *S. aureus* DSM 28763 biofilm formation by WBFB fractions. (a) Biofilm mass measured by CV assay and illustration of stained bacterial biofilm formed on polystyrene wells after incubation with WBFB fractions. (b) Log_10_ of biofilm cell numbers measured by CFU/mL after incubation with WBFB fractions. Biofilms were individually treated with extracts at MICs and compared to respective diluent controls. The bars represent means of three independent biological experiments with six technical replicates for CV and three for CFU assay ± SD; horizontal lines represent median. * denotes statistical differences between samples (*p* < 0.05). For abbreviations see Figure 1.

Three fractions showing antibacterial activity in the primary screening experiment were selected for further characterization of antibiofilm activity in *S. aureus* DSM 28763. Biofilms treated with acetone extract and tannin-like substances significantly reduced biomass by 83.0 and 95.9%, respectively. Even though dioxane lignin displayed antibacterial effect at 1.25 mg/mL (Table 5), it had only a mild effect on biofilm formation (i.e., biomass reduction of 33.0%) (Figure 4a). It should be noted that dioxane lignin aggregated within the biofilm complex when experiments were performed, and this effect could have led to the impairment of its activity. Still, all three fractions significantly decreased the biofilm mass when compared to their respective nontreated samples (Figure 4a). On average, a significant reduction of 4.3 log_10_ CFU/mL and 3.1 log_10_ CFU/mL was achieved by treating the biofilm with acetone extract and tannin-like substances, respectively (*p* < 0.05) (Figure 4b). Dioxane lignin treatment did not significantly reduce bacterial cells present in the biofilm (*p* > 0.05), relating to poor results observed in biomass assessment (Figure 4a). Overall, acetone extract and tannin-like substances at 1 and 3 mg/mL, respectively, are effective in inhibiting biofilm formation of *S. aureus* DSM 28763.

### Chemistry Profile of Bioactive Components

**Figure 5:**
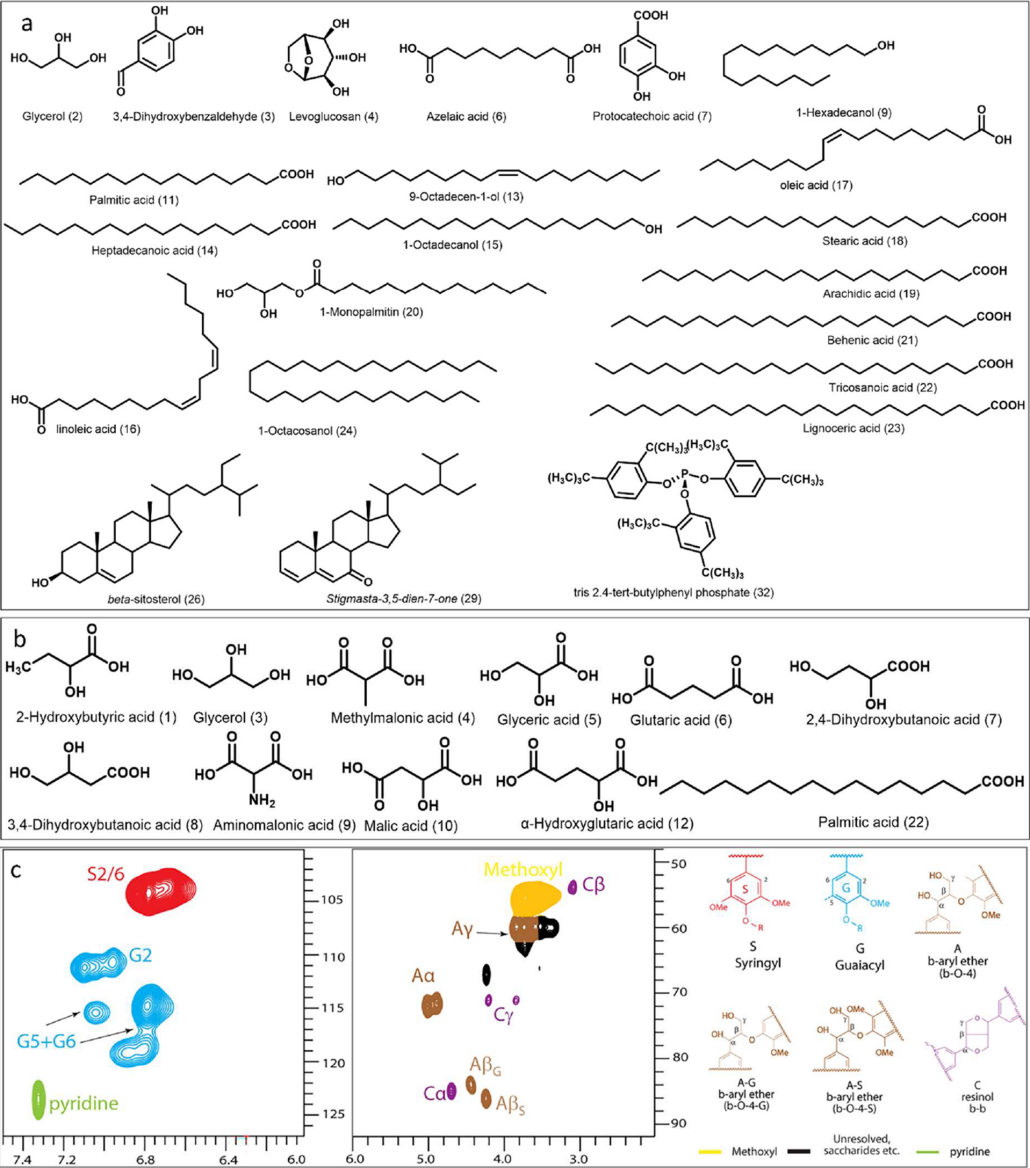
Chemical components detected from bioactive WBFB fractions. (a) Acetone extract, for the GC–MS spectrum, see Figure 6a. (b) Tannin-like. (c) Aromatic/unsaturated (δC/δH 100–127/6.0–7.5 ppm) and side chain (δC/δH 48–92/2.0–6.0 ppm) regions in the 2D HSQC NMR spectra of dioxane lignin in DMSO-*d*6/pyridine-*d*5 (v/v, 4/1). See Table S2 for signal assignments by comparison with the literature.^62^

This section outlines the chemistry profile of these bioactive compounds that showed biofilm formation inhibitory activity, particularly of the acetone extract, tannin-like, and dioxane lignin (Figure 5).

#### Acetone Extract

**Figure 6:**
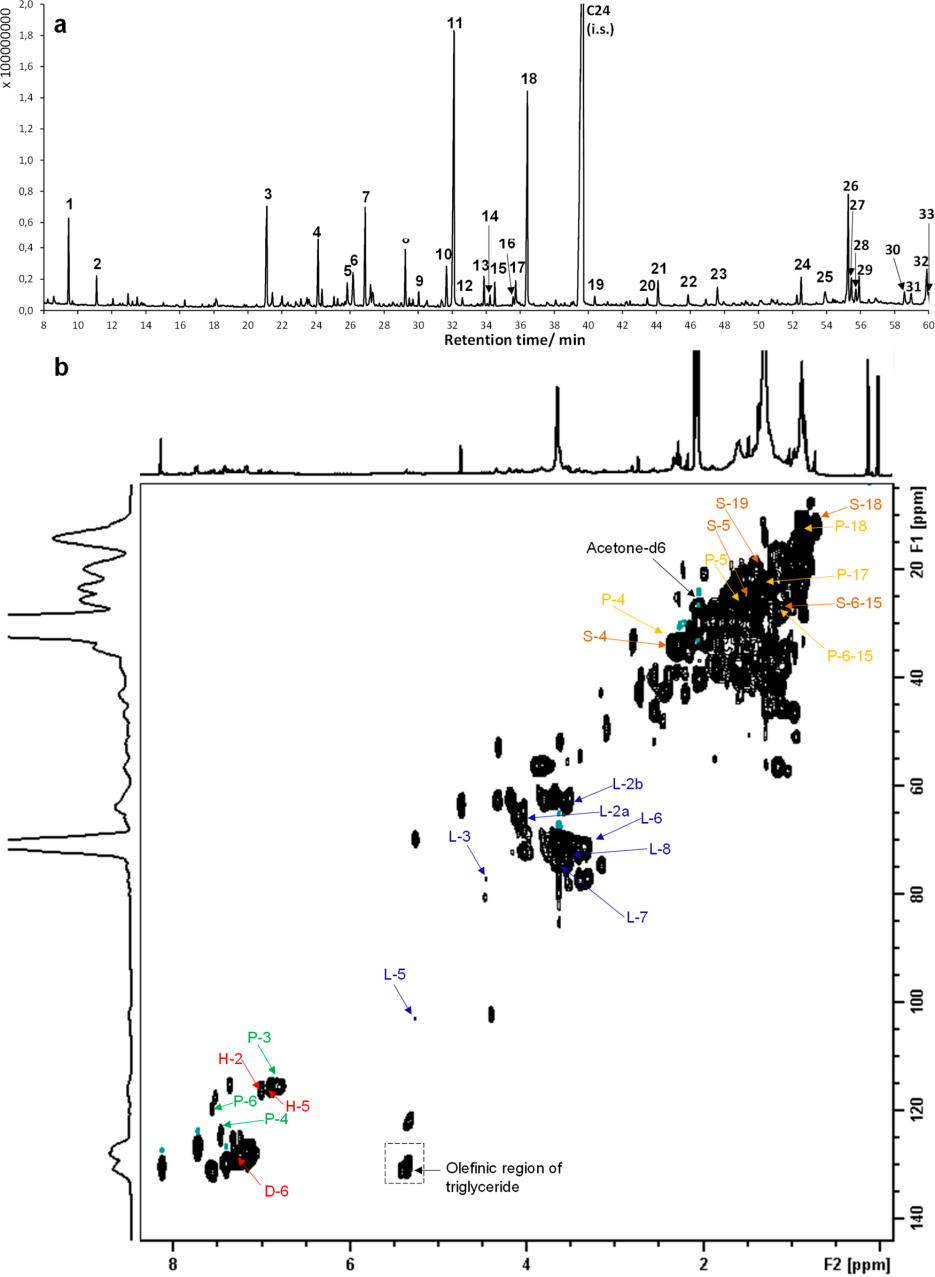
Chemical characteristics of the acetone extract. (a) GC–MS total-ion chromatogram of major peaks. Their trimethylsilyl derivatives of characteristic fragments and detected amounts (% w/w) are summarized in Table S1. (b) HSQC NMR spectrum (δC/δH, 5–142/0–8.3 ppm) in acetone–*d*6 (δC/δH, 29.92/2.05 ppm). The labels assign the signals from 3-hydroxybenzoic acid (H, red), levoglucosan (L, blue), protocatechuic acid (P, green), palmitic acid (P, yellow), and stearic acid (S, orange). The NMR spectra (^1^H and ^13^C) and chemical assignments of each authentic component are summarized at Figures S5 and S6–S9, respectively.

Significant signals from fatty acid triglycerides (e.g., glyceryl trioleate) (δC/δH, 130.46/5.35 ppm)^63^ were identified by NMR (Figures S4 and 6), indicating that triglycerides form the majority of the acetone extract, which cannot be detected by GC–MS because of its detection limit on analyzing extractive fractions of the pitch deposits.^64^ Other unsaturated fatty acids like linoleic acid and oleic acid (Figures 5a and 6a) were identified from GC–MS. Antibacterial and biofilm formation inhibitory effect of unsaturated triglycerides and fatty acids is well documented in the literature. In particular, in a study by Lee and colleagues, unsaturated fatty acids inhibited *S. aureus* biofilms, whereas saturated fatty acids, including oleic and stearic acid, lacked any significant effect,^65^ reviewed in ref (66). Therefore, we consider them as major contributors to the observed strong antibacterial activity. Analyses by GC–MS revealed roughly 10 wt % of the acetone extract, in which the major identified compounds were 3,4-dihydroxybenzaldehyde, levoglucosan, protocatechuic acid, palmitic acid, and stearic acid. Protocatechuic acid (0.7 wt %) is known to possess multiple biological activities. This natural phenolic acid on its own, as well as through synergy with antibiotics, have been reported to inhibit a broad spectrum of pathogenic species, including *S. aureus*.^67,68^ Antibacterial activity of protocatechuic acid was also confirmed in our study where we observed partial inhibition of bacterial growth at a concentration of 2 mg/mL, whereas levoglucosan (2 wt %) showed no activity (Table 5). Furthermore, another major compound 3,4-dihydroxybenzaldehyde has been previously shown to be active against *E. coli*, *S. typhimurium*, and *S. aureus* by the disk diffusion assay,^69^ and therefore, potentially contributes in the bioactivity observed in our study, albeit not tested.

#### Tannin-like Substances

Only less than 20 wt % of tannin-like substances were identified by GC–MS (Table S3). This may be due to the fact that the molecular weight (MW) of tannin ranges from 500 to 3000 Da, and its trimethylsilyl derivatives are often beyond the detection limit of GC–MS. Demonstrated major components were 7.2 wt % of dicarboxylic acids (methylmalonic acid, glutaric acid, aminomalonic acid, malic acid, and α-hydroxyglutaric acid), 1.1 wt % of fatty acids (palmitic acid), 4.6 wt % of sugar acid (glyceric acid, 2,4-dihydroxybutanoic acid, and 3,4-dihydroxybutanoic acid), and 3.1 wt % 2-hydroxybutyric acid (Figure S10). Out of these, malic acid has been previously shown to kill *S. aureus*, *E. faecalis*, *E. coli*, and *P. aeruginosa* with MIC ranging between 500 and 1000 μg/mL^70^ and eradicated *E. faecalis* biofilms.^71^ It is challenging to interpret chemical composition of these unidentified intense cross-peaks (Figure 6b) without any mass spectrum fingerprints. Further elucidation using high resolution-liquid chromatography mass spectrometry (HR-LCMS) may succeed in revealing the unknown fractions of the acetone extracts and tannin-like; however, this is out of the scope of this present study.

#### Dioxane Lignin

Solution-state 2D NMR spectroscopy was applied to obtain the chemical profile of dioxane lignin. The S/G ratio (1.2) and the relative interunit linkage ratio (A:C, 85:14) between β-O-4′ aryl ethers (A′/A) and resinols (C) were obtained by integration of ^1^H–^13^C correlation contours in the corresponding HSQC spectra, as shown in Figure 5c, which is similar to lignin obtained from the willow inner bark through enzyme treatment (ratios of S/G and A/C are 0.9 and 80:17, respectively).^62^ These unidentified signals (Figure S11) could be associated with the coextracted impurities (e.g., protein and polysaccharides). Understanding the exact lignin moieties (or lignin precursors) that are responsible for this shown bioactivity is out of the scope of this present study.

In conclusion, we report here the first potential use of wood bark-derived materials for antibacterial and antibiofilm wound dressings to be utilized in chronic infected wound care. We show that yarns containing at least 50% of WBFBs significantly inhibit biofilm formation by *S. aureus* strains isolated from infected wounds, including a multidrug-resistant strain. Although *S. aureus* plays a critical role in wound infections, focus on a single bacterial species alone is a limitation of this study. We determined the contribution of each recovered fraction in the observed activity by deconstructing the bioactive WBFBs. Dioxane lignin is the major identified contributor to the antibacterial activity against planktonic bacteria, whereas tannin-like substances and acetone extracts (presumably unsaturated fatty acid components) play an important role in antibiofilm activity of the material. WBFB-based materials are affordable and biodegradable and comply with the principles of green chemistry. Importantly, similar types of fiber bundles can be recovered and extracted from the bark of other fast-growing trees, such as eucalyptus and poplar. The outcome of this study is encouraging and justifies further development of WBFB-based wound dressing materials, e.g., generation a wound dressing prototype and testing it for biocompatibility and activity in infected wound animal models.

## Supporting Information Available

Details of antibiofilm and antibacterial assessment of extracted components, the photographs of the fractionated components and different forms of WBFBs, biofilm formation assessment, chemical characteristics of the studied WBFB/lyocell mixtures, and NMR spectrum of the model compounds (including glyceryl trioleate, levoglucosan, protocatechuic acid, palmitic acid, and stearic acid) (PDF)
