# Supplementary material for: Willow Bark-Derived Material with Antibacterial and Antibiofilm Properties for Potential Wound Dressing Applications
Source: J Agric Food Chem. 2023 Apr 27;71(44):16554–67. doi: 10.1021/acs.jafc.3c00849 (PMC10636761; doi:10.1021/acs.jafc.3c00849)
Supplement: Supplementary file 1 — jf3c00849_si_001.pdf [file jf3c00849_si_001.pdf]

Supporting Information

Willow bark-derived material with antibacterial and antibiofilm properties for potential wound dressing applications

Jinze Dou[1*¤]; Polina Ilina[2*]; Cristina D. Cruz[2]; Denise Nurmi[1]; Paula Zegarra Vidarte[2]; Marja Rissanen[1]; Päivi Tammela[2]; Tapani Vuorinen[1]

[1]Department of Bioproducts and Biosystems, School of Chemical Engineering, Aalto University, Finland

[2]Drug Research Program, Division of Pharmaceutical Biosciences, Faculty of Pharmacy, University of Helsinki, Finland

*Equal contribution

¤Corresponding author: J. Dou Telephone: +358 413115001. E-mail: jinze.dou@aalto.fi

Details of antibiofilm and antibacterial assessment of extracted components

Antibacterial activity of the individual components recovered from WBFB.

Briefly, a loopful of bacteria from the overnight MHA plate culture was transferred into sterile 0.9% saline solution and bacterial suspension with concentration of $1x10^6$ colony forming units (CFU)/mL was prepared in cation-adjusted Mueller Hinton broth (CAMHB, BD). The test samples were diluted in CAMHB to 2x final concentration in strip tubes. 100 µL of the sample and control dilutions were transferred onto a clear sterile polystyrene 96-well plate (Thermofisher Scientific) in triplicate followed by 100 µL of bacterial suspension. Ciprofloxacin at the minimum inhibitory concentration (MIC, 0.5 µg/mL) was used as a positive control. No bacterial suspension was added into the minimum control (only CAMHB). The assay plate was incubated at 37 °C with a shaking speed of 500 rpm (PST-60HL-4 Thermoshaker). Absorbance at 620 nm was measured at 0 and 24 h timepoints.

Assessment of biofilm formation by wound-isolated *S. aureus* strains.

Briefly, the formed biofilm was washed once with phosphate-buffered saline (1x PBS;137 mM NaCl, 2.7 mM KCl, 1 mM Na_2HPO_4; and 2 mM KH_2PO_4; pH 7.4). For CFU assays, biofilm cells were suspended in 1x PBS by vigorous pipetting, followed by appropriate 10-fold dilutions. Then, aliquots of 10 µL were drop-plated on TSA plates and CFU enumerated after 24 h of incubation at 37 °C. For biomass, the washed biofilm was fixed with 100% ethanol for 5 min, air-dried for 10 min and stained with 0.01% of crystal violet (CV, Sigma-Aldrich). After 30 min of incubation biofilm was washed with sterile water and air-dried for 30 min. Finally, 96% w/v of ethanol was added, and stained biofilm was resuspended by vigorous pipetting. CV absorbance measurements at 570 nm were performed using Multiskan GO (Thermo Fisher Scientific). For metabolic activity assessment, resazurin solution (4 µg/mL, Sigma-Aldrich) was added into washed biofilms. Plates were then incubated at 25 °C for 20 min in the dark. A multimode microplate reader (Varioskan LUX, Thermo Fisher Scientific) was used to measure the relative fluorescence units (RFU, λex=530 nm and λem=590 nm).

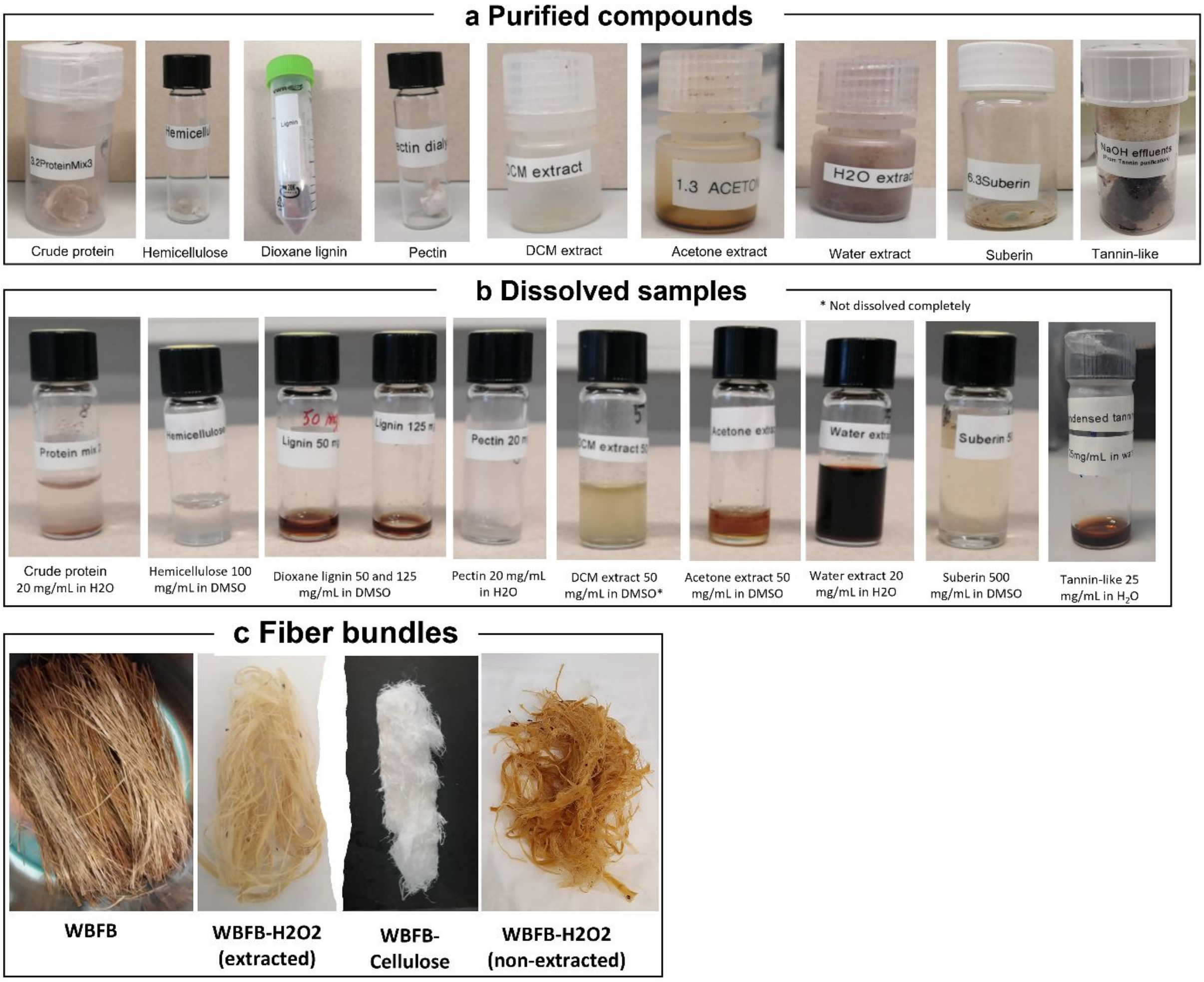

Fig. S1 The photographs of the fractionated components and different forms of willow bark fiber bundles (WBFB). a Purified component. **b** Purified components in their dissolved form for bioactivity experiments. **c** Fiber bundles that have been used in this study.

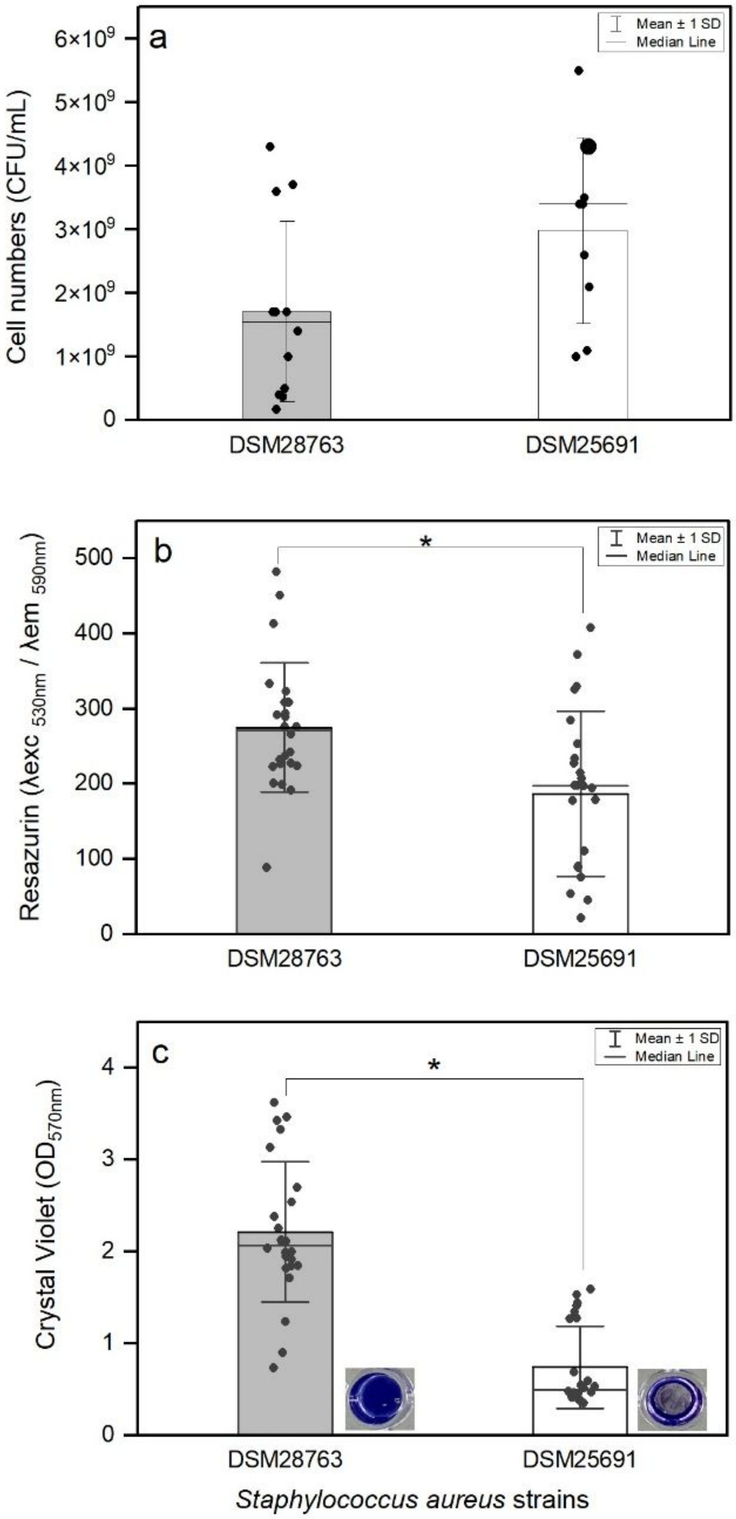

Fig. S2 Biofilm formation assessment of *S. aureus* DSM 28763 and DSM 25691. **a** Cell numbers measured by CFU/ml. **b** Metabolically active cells measured by resazurin fluorescence (λ_{ex}=530 nm and λ_{em}=590 nm). **c** Biofilm mass measured by crystal violet (CV) absorbance (OD_{570nm}) and illustration of stained bacterial biofilm formed on polystyrene wells. Mean of four independent biological experiments (n: 6) for resazurin and CV assays. Mean of three independent experiments for cell numbers determination (n: 4). *Denotes for significant differences ($p<0.05$).

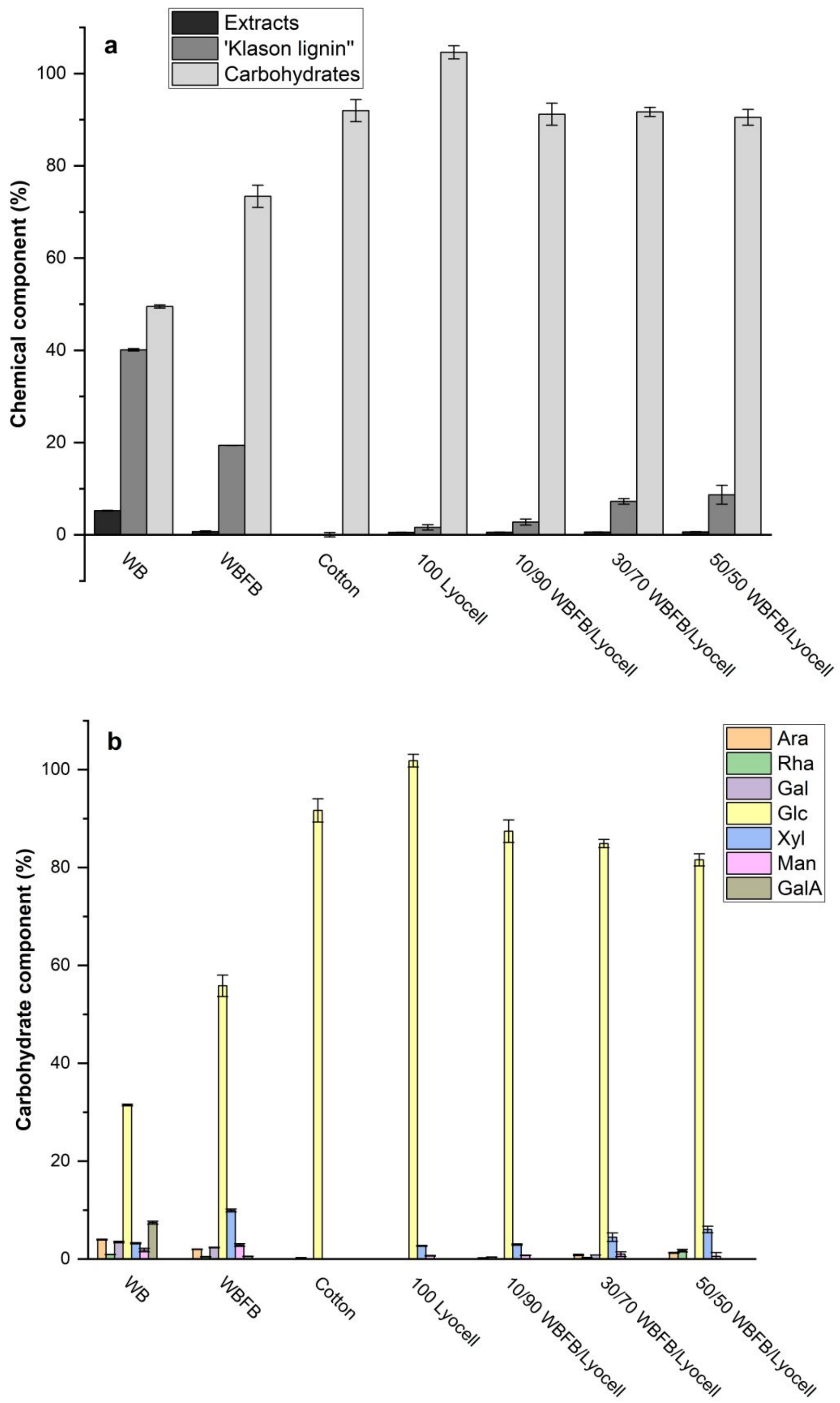

Fig. S3 Chemical characteristics of the studied WBFB/Lyocell (Trademark: Tencel) mixtures in comparison with the original willow bark (WB), willow bark fiber bundles (WBFB), cotton, and Lyocell. a Overall chemical composition (% of the original dry mass); **b** Carbohydrate composition (% of anhydro sugars in the original dry mass). Abbreviations: Arabinose (Ara), rhamnose (Rha), galactose (Gal), glucose (Glc), xylose (Xyl), mannose (Man), galacturonic acid (GalA).

Table S1. Chemical composition of detectable components (10.6 ± 0.6 wt % detected) as weight percentage (wt %) of fractionated acetone extracts from WBFB. MS spectra were referenced with the NIST chemistry book [N].

Retention time, min	Peak number	Compound, detected as methyl ester (Me) and/ or TMS ester/ ether (TMS) derivatives	Amount, % (w/w)	Characteristic fragments m/z	MW of TMS derivatives	Reference
9.46	1	Unidentified	0.4 (0.13)	193; 178; 149; 135; 116	–	–
11.107	2	Glycerol (3TMS ethers)	0.1 (0.005)	218; 205; 147; 133; 117; 103; 73	308.6372	[N]
21.111	3	3.4-Dihydroxybenzaldehyde (2TMS ethers)	0.9 (0)	282; 267; 193; 73	282.111	[N]
24.128	4	Levoglucosan (3TMS ethers)	0.4 (0.001)	333; 217; 204; 147; 129; 73	378.684	[N]
25.832	5	Unidentified	0.1 (0.01)	295; 251; 204; 147; 129	–	–
26.174	6	Azelaic acid (2TMS esters)	0.3 (0.02)	317; 201; 149; 117; 73; 55	332.5832	[N]
26.887	7	Protocatechoic acid (2TMS ethers; TMS ester)	0.7 (0.02)	370; 355; 311; 281; 223; 193; 73	370.6635	[N]
29.246	8	Monosaccahride (5TMS ethers)	0.3 (0)	217; 204; 191; 147; 129; 73	541.0615	[N]
30.021	9	1-Hexadecanol (TMS ether)	0.1 (0)	299; 111; 103; 75; 73	314.3	[N]
31.666	10	Monosaccahride (5TMS ethers)	0.3 (0)	217; 204; 191; 147; 129; 73	541.0615	[N]
32.112	11	Palmitic acid (TMS ester)	2.5 (0.01)	313; 145; 132; 129; 117; 75; 73	328.6052	[N]
32.597	12	Unidentified	0.0 (0.01)	339; 221; 194; 179; 140; 116	–	–
33.868	13	9-Octadecen-1-ol (TMS ether)	0.2 (0.01)	340; 325; 297; 129; 96; 75	340.6590	[N]
34.225	14	Heptadecanoic acid (TMS ester)	0.1 (0)	327; 145; 132; 117; 75; 73	342.6318	[N]
34.51	15	1-Octadecanol (TMS ether)	0.1 (0)	327; 129; 111; 97; 75	342.6748	[N]
35.583	16	Linoleic acid (TMS ester)	0.1 (0.01)	352; 337; 262; 220; 129; 73	352	[N]
35.735	17	Oleic acid (TMS ester)	0.2 (0.02)	354; 339; 264; 145; 129; 117; 75	354.6425	[N]
36.421	18	Stearic acid (TMS ester)	1.7 (0.04)	341; 145; 132; 129; 117; 75; 73	356.6584	[N]
39.705		C24	9.3	–	–	–
40.38	19	Arachidic acid (TMS ester)	0.1 (0)	384; 369; 201; 145; 132; 117; 73	384.7115	[N]
43.471	20	1-Monopalmitin (2 TMS ethers)	0.1 (0.09)	459; 371; 239; 147; 129; 73	474.8649	[N]
44.098	21	Behenic acid (TMS ester)	0.2 (0)	412; 397; 145; 132; 117; 73	412.7647	[N]
45.869	22	Tricosanoic acid (TMS ester)	0.1 (0.01)	412; 397; 145; 132; 117; 73	426	[N]
47.593	23	Lignoceric acid (TMS ester)	0.1 (0.03)	440; 425; 201; 145; 132; 117; 73	440.8178	[N]
52.508	24	1-Octacosanol (TMS ether)	0.2 (0.03)	467; 103; 97; 91; 75; 57	482.48	[N]
53.921	25	Unidentified	0.1 (0.03)	481; 422; 382; 343; 255; 207; 145; 129	–	–
55.275	26	β-Sitosterol (TMS ether)	0.6 (0.27)	486; 471; 396; 381; 357; 129; 73	486.8878	[N]
55.461	27	Unidentified	0.1 (0.09)	495; 207; 111; 75	–	–
55.723	28	Unidentified	0.1 (0)	480; 257; 218; 111; 57	–	–
55.907	29	Stigmasta-3.5-dien-7-one	0.2 (0.02)	410; 269; 187; 174; 161; 43	410.35	[N]
58.573	30	Unidentified	0.1 (0.01)	523; 257; 207; 111; 69	–	–
58.972	31	Unidentified	0.1 (0)	431; 285; 257; 207; 147; 111; 69	–	–
59.892	32	Tris 2.4-tert-butyphenyl phosphate	0.2 (0.02)	648; 316; 191; 147; 57	662.9210	[N]
59.988	33	Unidentified	0.0 (0.01)	648; 500; 395; 207; 161; 129		
sum			10.6 (0.57)			

Table S2. Assignments of the ^{13}C–^{1}H correlation peaks in the 2D HSQC spectra of the dioxane lignin from WBFB.

Label	δC/δ H (ppm)	Assignment
$C_β$	53.6/3.09	$C_β$−$H_β$ in β−β′ resinol substructures **(C)**
$−OCH_3$	55.2/3.73	C−H in methoxyls
$A_γ$	59.7/3.76 and 59.6/3.53	$C_γ$−$H_γ$ in γ-hydroxylated β-O-4′ substructures **(A)**
$C_γ$	70.7/4.21 and 70.8/3.88	$C_γ$−$H_γ$ in β−β′ resinol substructures **(C)**
$A_{β(G)}$	83.7/4.46	$C_β$−$H_β$ in β-O-4′ substructures **(A)** linked to a G- unit
$C_α$	84.8/4.72	$C_α$−$H_α$ in β−β′ resinol substructures **(C)**
$A_{β(S)}$	85.7/4.25	$C_β$−$H_β$ in β-O-4′ substructures **(A)** linked to a S- unit
$S_{2/}S_6$	103.9/ 6.8	C_2−H_2 and C_6−H_6 in syringyl units **(S)**
G_2	110.7/7.11 and 110.2/6.98	C_2−H_2 in guaiacyl units **(G)**
G_5/G_6	115.0/7.07; 114.4/6.81 and 118.8/6.92	C_5−H_5 and C_6−H_6 in guaiacyl units **(G)**

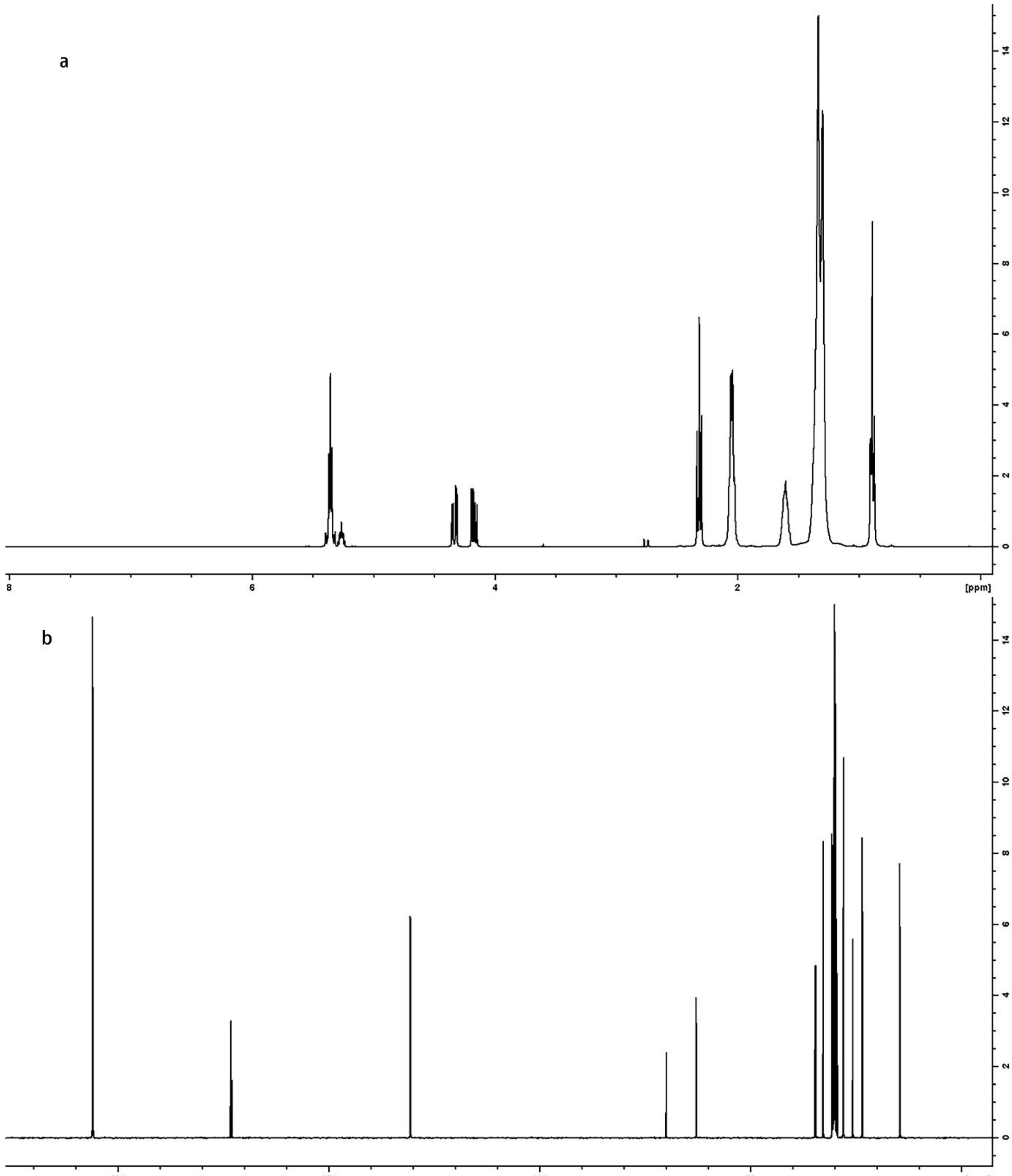

a
b
[ppm]
8
6
4
2
0
2
4
6
8
10
12
14
200
150
100
50
[ppm]

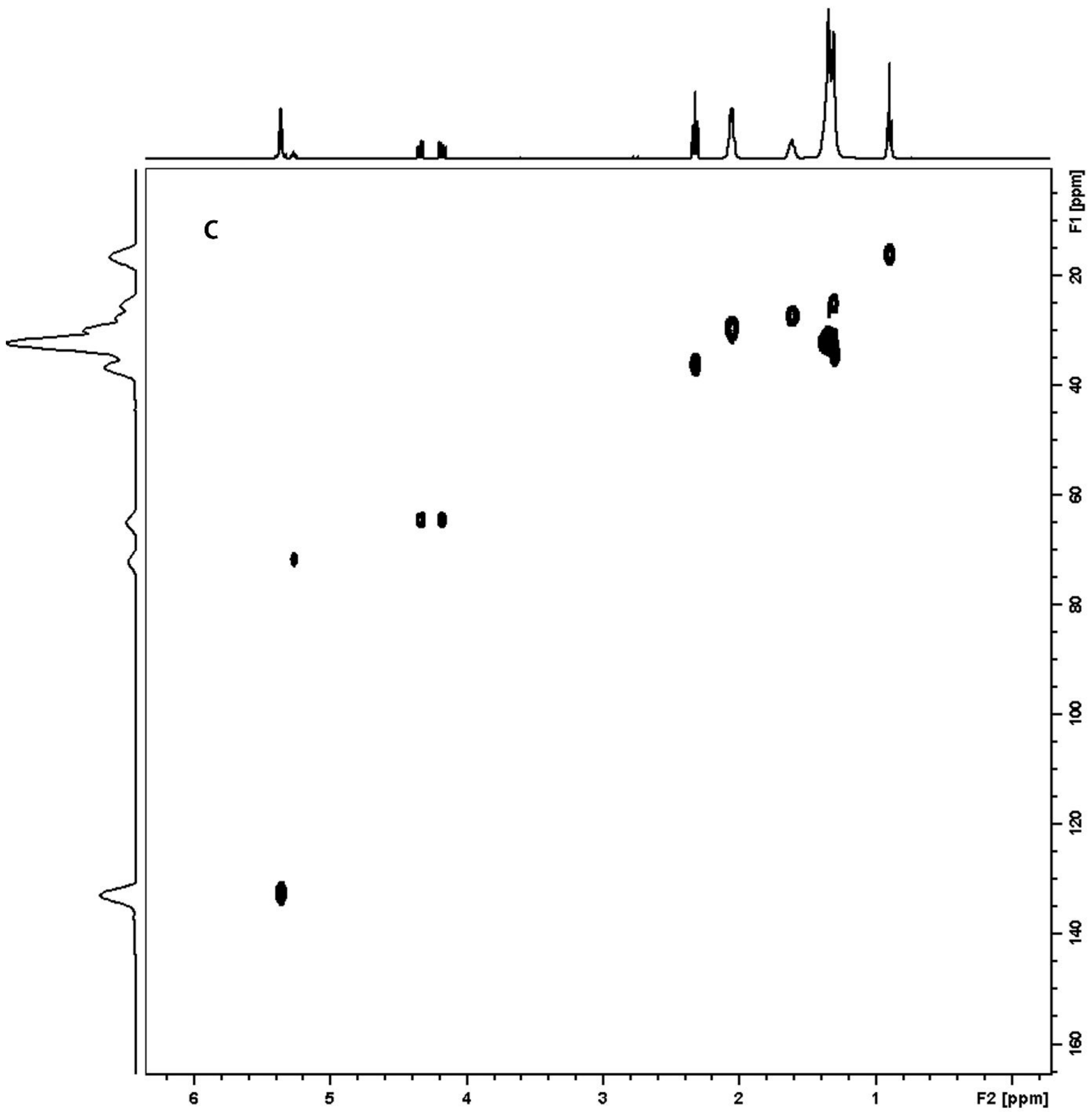

Fig. S4 NMR spectrum of the authentic glyceryl trioleate in acetone–*d6* (δC/δH, 29.92/2.05 ppm). **a** ^{1}H. **b** ^{13}C. **c** 2D ^{1}H–^{13}C HSQC.

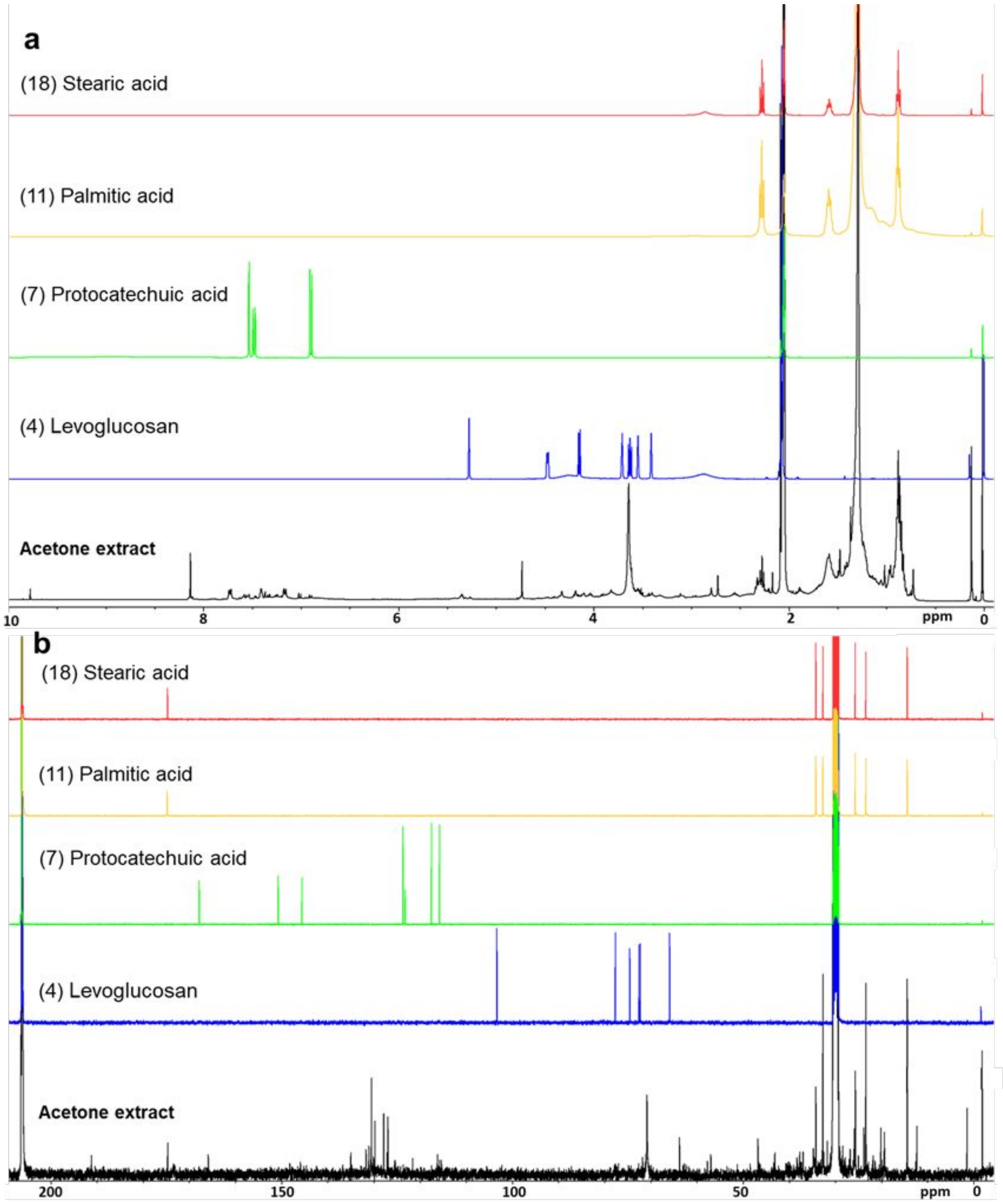

Fig. S5 Overlapped NMR spectrum of the acetone extract and its authentic components (i.e. (4) levoglucosan; (7) protocatechuic acid; (11) palmitic acid and (18) stearic acid) (Fig. 5a) in acetone-*d6*. a ^{1}H. **b** ^{13}C. The labels assign the signals from Levoglucosan (L, blue), Protocatechuic acid (P, green), Palmitic acid (P, yellow), and Stearic acid (S, orange).

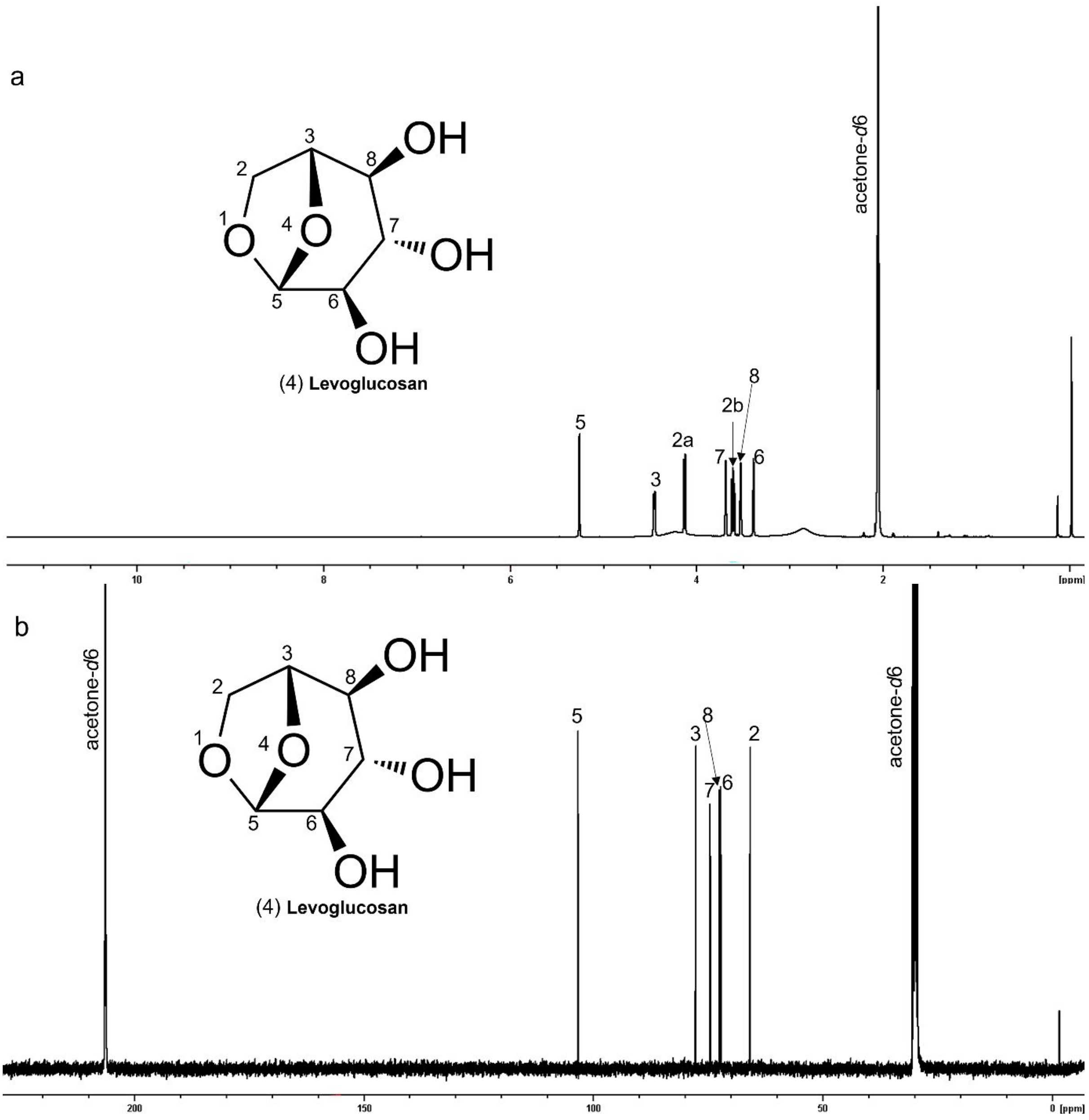
a
3
8
OH
2
1
4
O
O
7
OH
5
6
OH
(4) **Levoglucosan**
acetone-*d6*
5
3
2a
7
2b
8
6
10
8
6
4
2
[ppm]
b
acetone-*d6*
3
8
OH
2
1
4
O
O
7
OH
5
6
OH
(4) **Levoglucosan**
5
3
8
2
7
6
acetone-*d6*
200
150
100
50
0 [ppm]

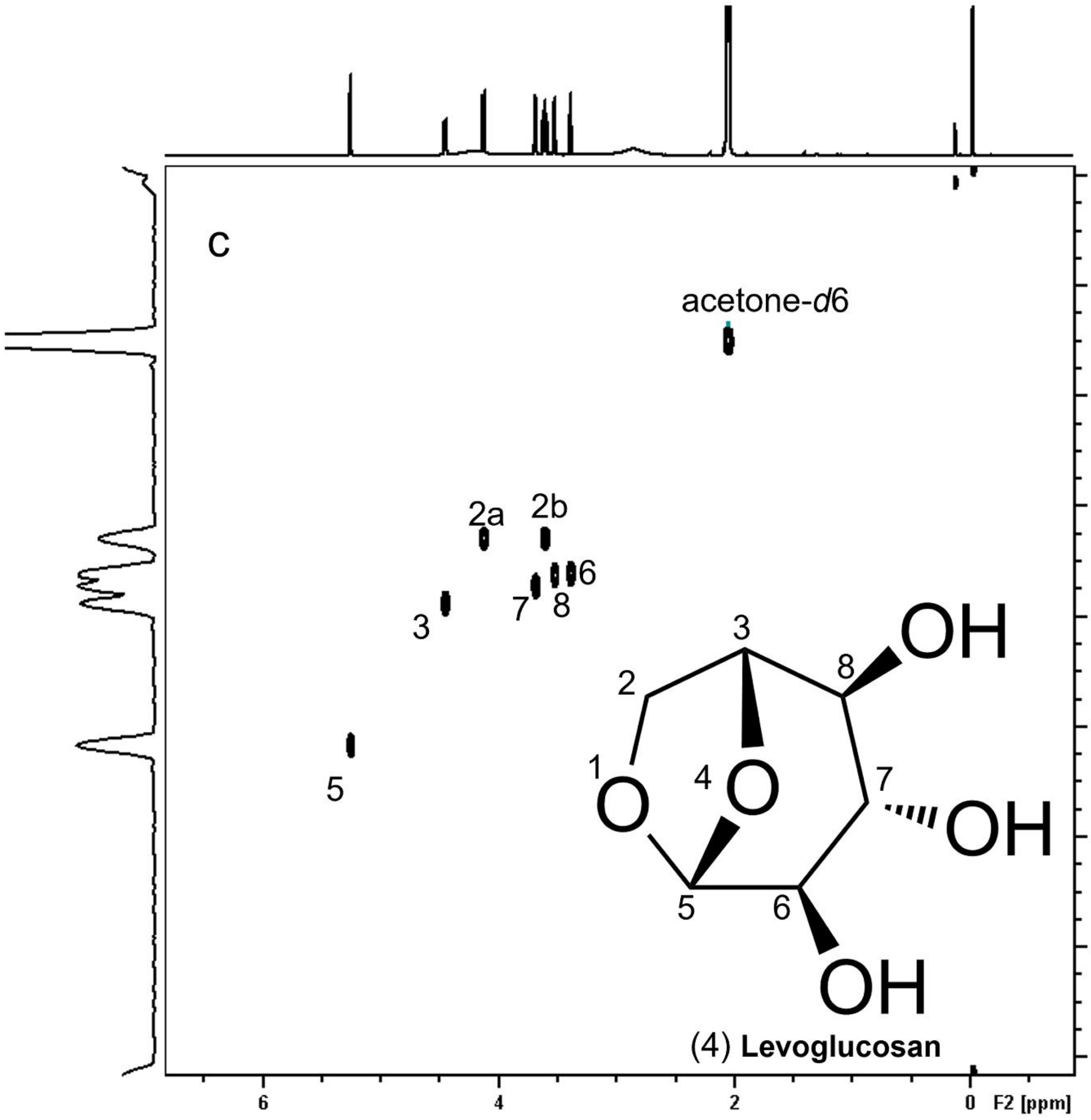

Fig. S6 NMR spectrum of the authentic (4) levoglucosan (Fig. 5) in acetone–*d6* (δC/δH, 29.92/2.05 ppm). **a** ^{1}H. **b** ^{13}C. **c** 2D ^{1}H–^{13}C HSQC.

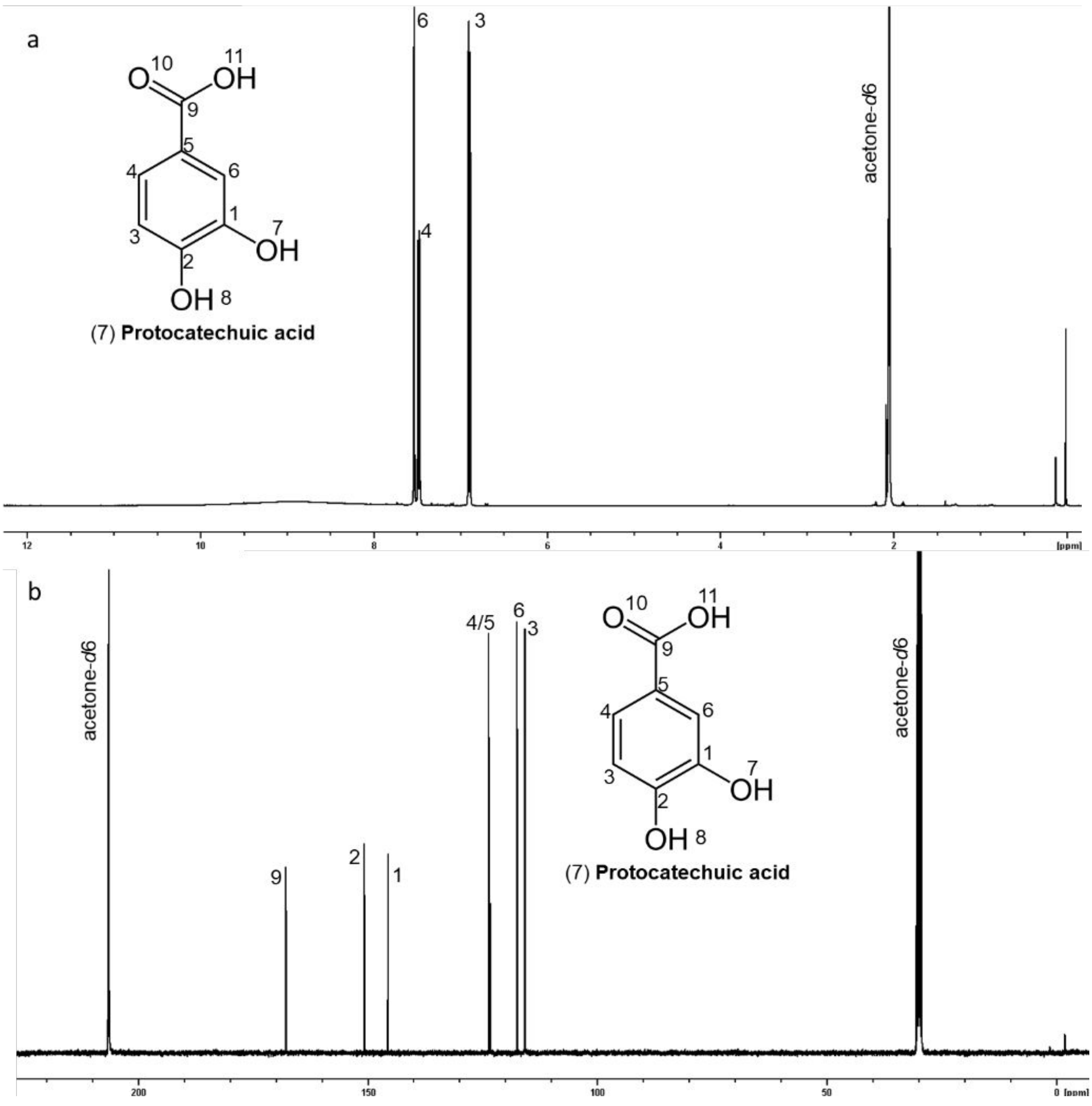
a
(7) Protocatechuic acid
acetone-d6
[ppm]
b
4/5
acetone-d6
(7) Protocatechuic acid
0 [ppm]

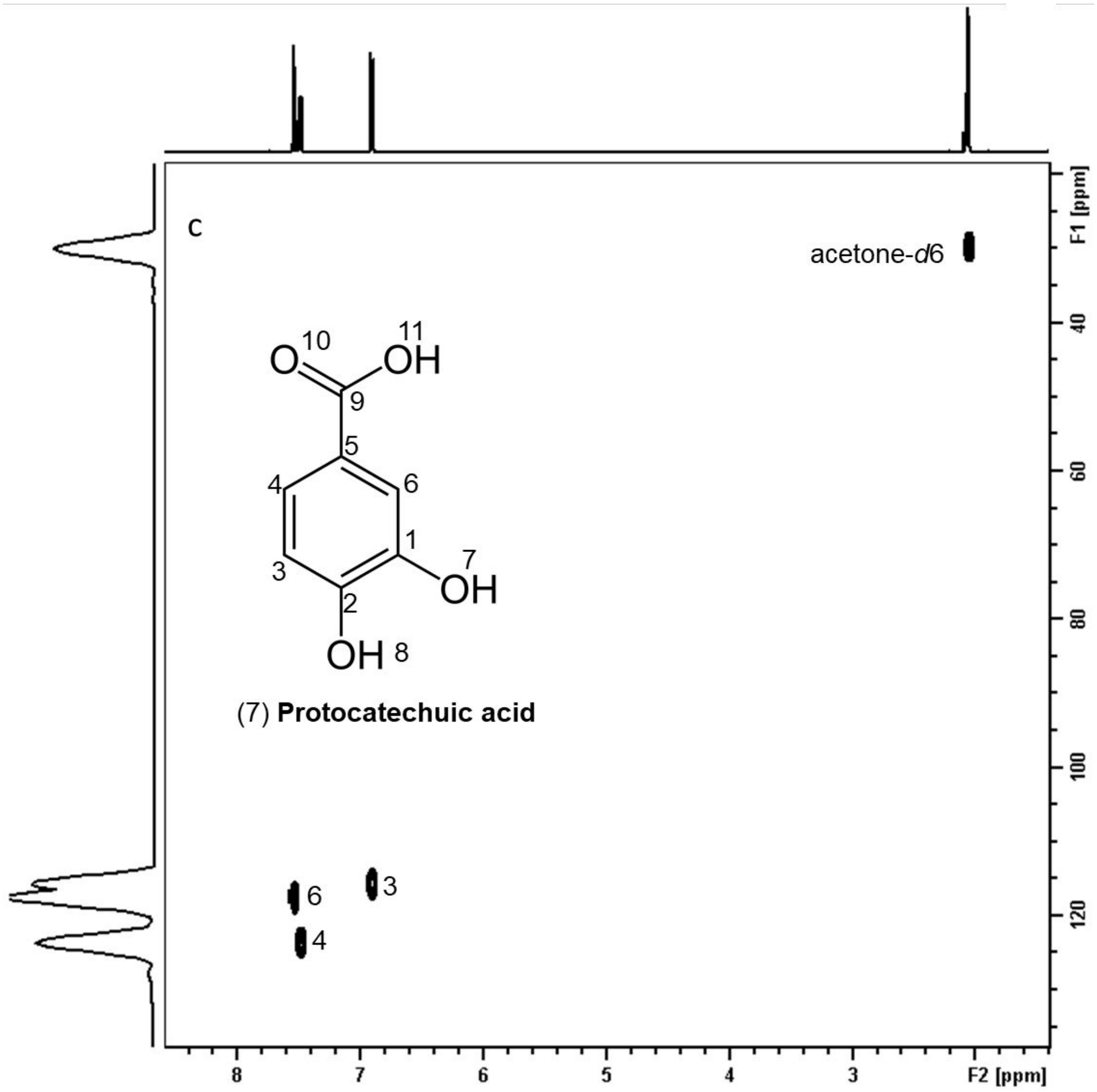

Fig. S7 NMR spectrum of the authentic (7) protocatechuic acid in acetone–*d6* (δC/δH, 29.92/2.05 ppm). a ^{1}H. **b** ^{13}C. **c** 2D ^{1}H–^{13}C HSQC.

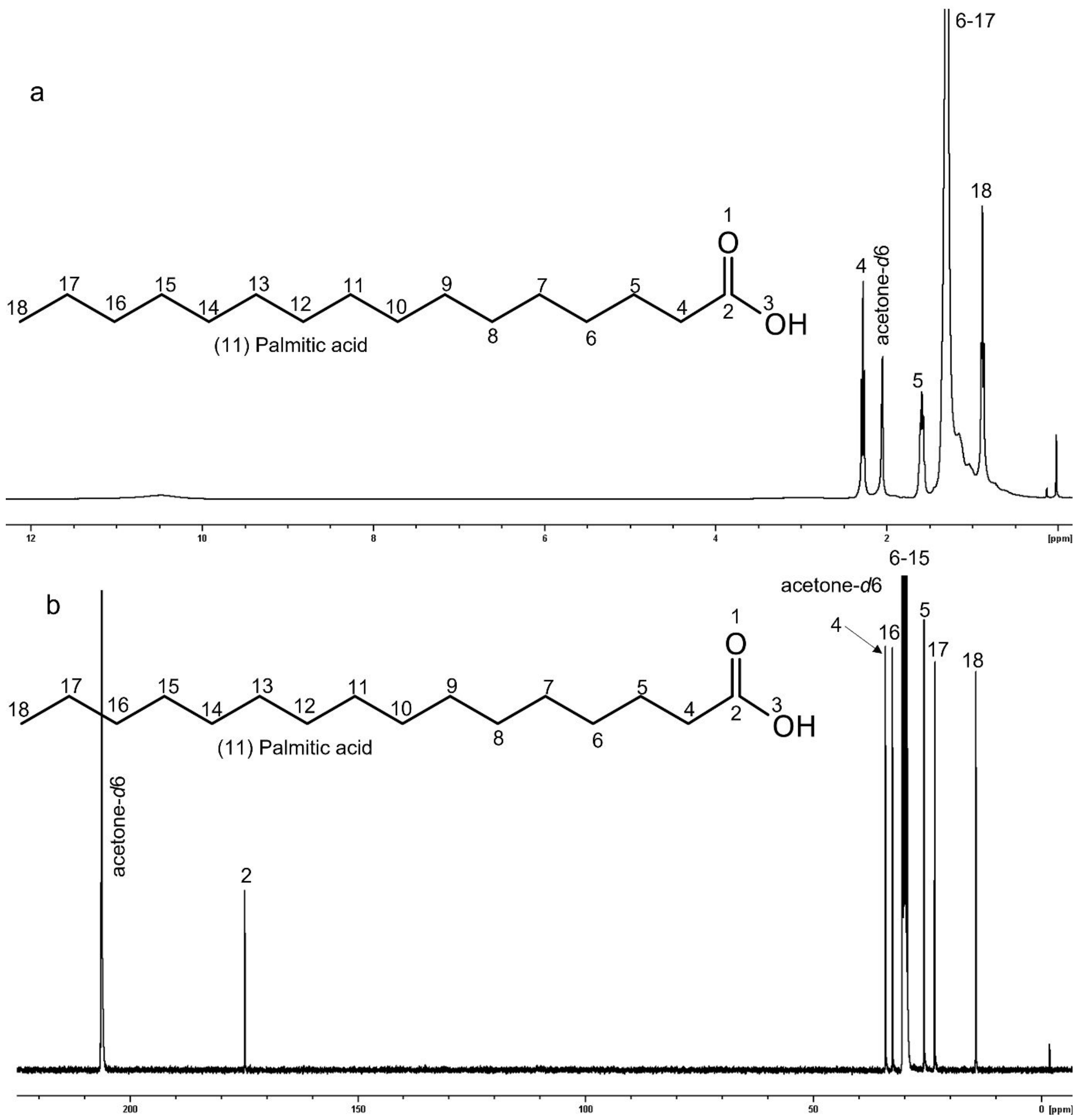

a
6-17
18
1
O
4
acetone-d6
17
15
13
11
9
7
5
18
16
14
12
10
4
2
3
OH
8
6
(11) Palmitic acid
5
12
10
8
6
4
2
[ppm]
b
6-15
acetone-d6
1
O
4
16
5
17
18
17
15
13
11
9
7
5
18
16
14
12
10
4
2
3
OH
8
6
(11) Palmitic acid
acetone-d6
2
200
150
100
50
0 [ppm]

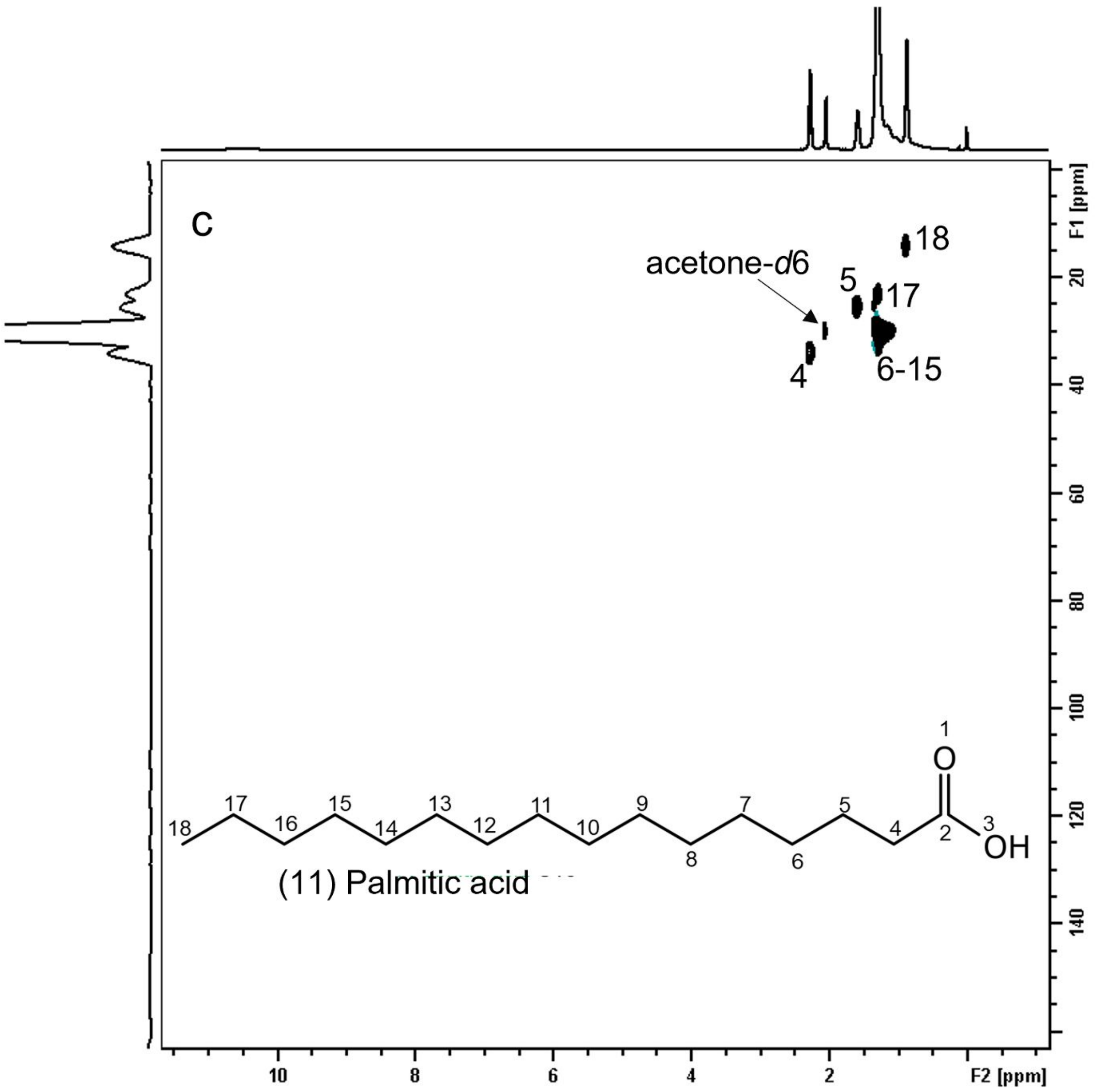

Fig. S8 NMR spectrum of the authentic (11) palmitic acid in acetone–*d6* (δC/δH, 29.92/2.05 ppm). a ^{1}H. **b** ^{13}C. **c** 2D ^{1}H–^{13}C HSQC.

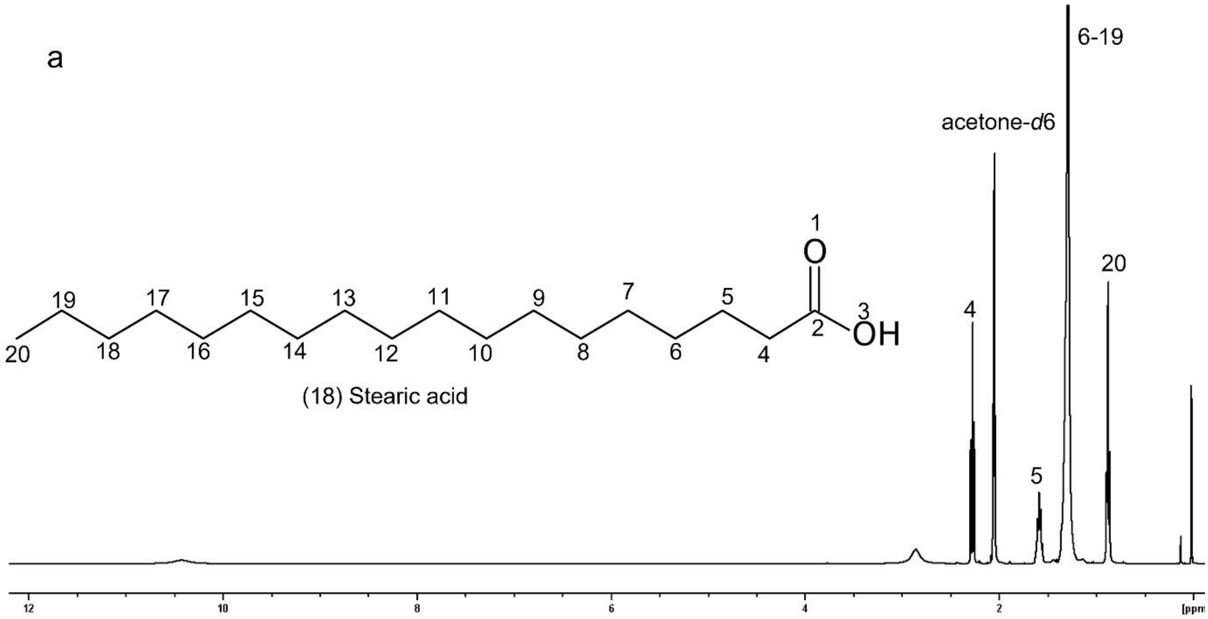
a
6-19
acetone-d6
1
O
20
19
17
15
13
11
9
7
5
4
3
2
OH
20
18
16
14
12
10
8
6
4
(18) Stearic acid
5
12
10
8
6
4
2
[ppm

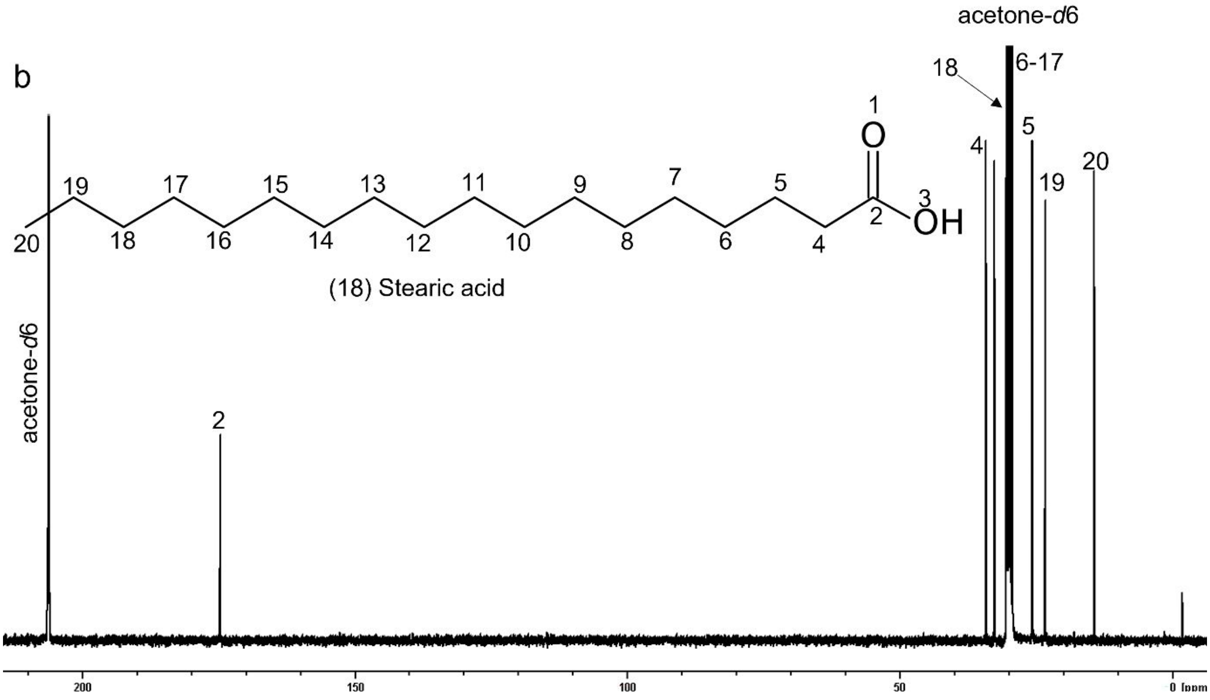
acetone-d6
b
18
6-17
1
O
4
5
20
19
17
15
13
11
9
7
5
19
3
2
OH
20
18
16
14
12
10
8
6
4
(18) Stearic acid
acetone-d6
2
200
150
100
50
0 [ppm

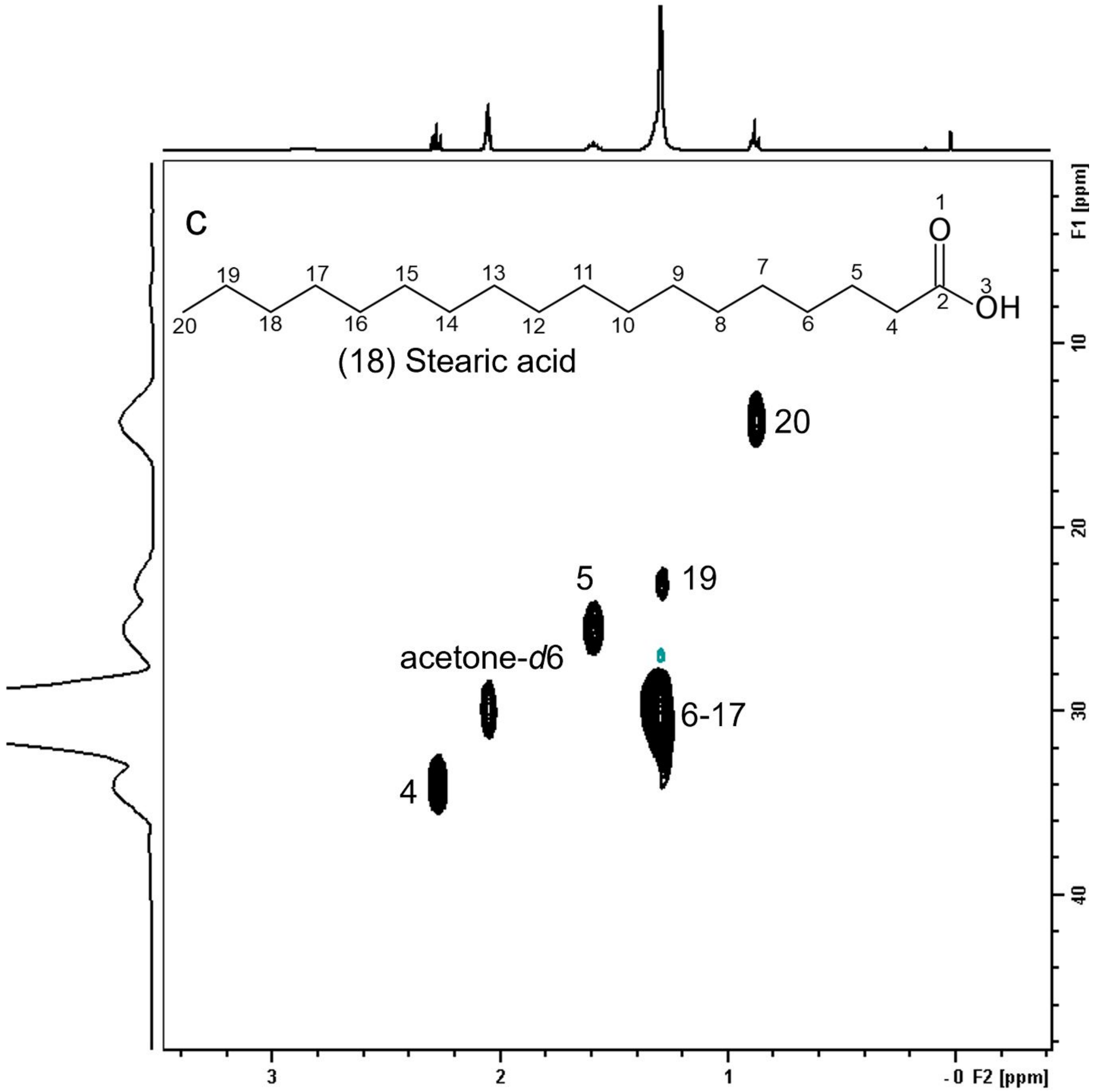

Fig. S9 NMR spectrum of the authentic (18) stearic acid in acetone–*d6* (δC/δH, 29.92/2.05 ppm). a ^{1}H. **b** ^{13}C. **c** 2D ^{1}H–^{13}C HSQC.

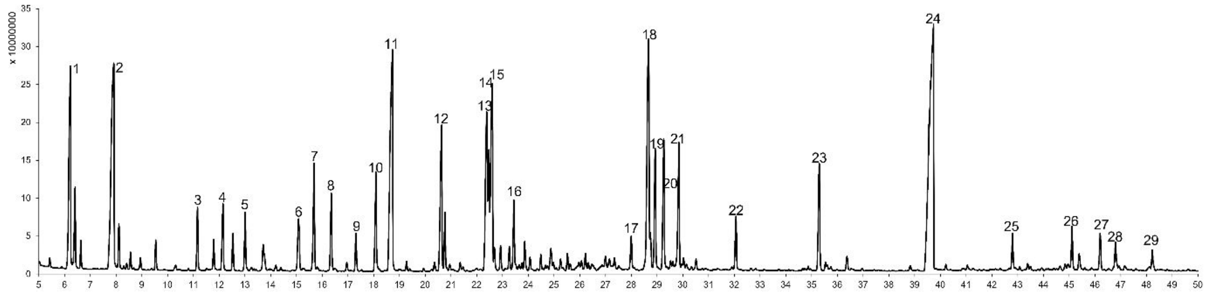

Fig. S10 GC–MS total-ion chromatogram of the major peaks of tannin-like components obtained from WBFB (**Fig. 1**). Their characteristic m/z are summarized at **Table S3**.

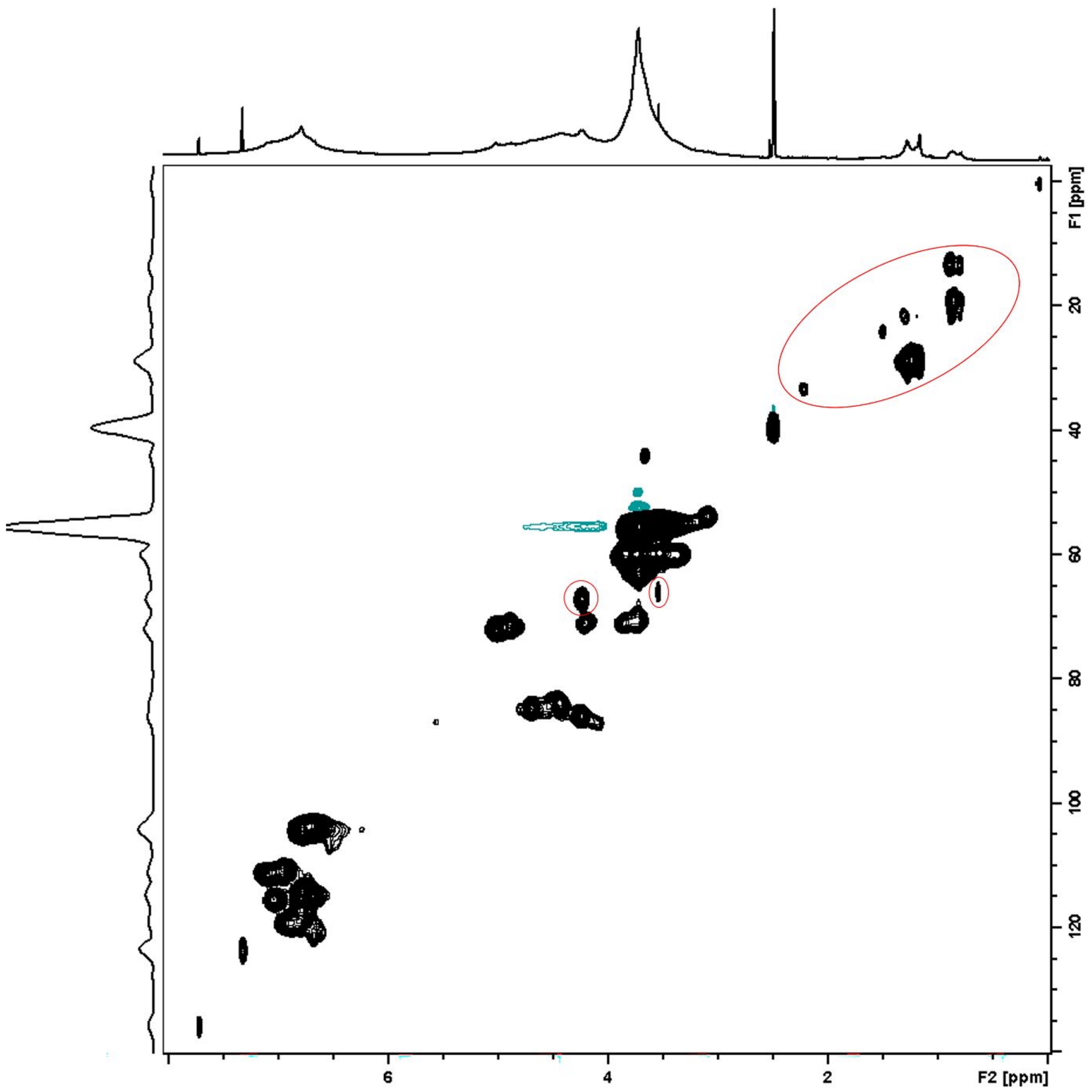

Fig. S11. **Full 2D HSQC NMR spectra of dioxane lignin in DMSO-d6/pyridine-d5 (v/v, 4/1).** The unidentified peaks are highlighted in red.

Table S3. Chemical composition of tannin-like substances (47.8 wt % detected) that is obtained from WBFB by 0.1M NaOH treatment as weight percentage (wt %) of the analysed tannin-like fraction. MS spectra were reference with the NIST chemistry book [N].

Retention time, min	Peak number	Compounds, detected as methyl ester (Me) and/or TMS ester/ether (TMS) derivatives	Amount, % (w/w)	Characteristic fragments m/z	MW of TMS derivatives	DB/ Reference
39.593	24	Tetracosane (C24)	3.3	338; 99; 85; 71; 57; 43	338.6538	[N]
6.193	1	2-Hydroxybutyric acid, 2 TMS derivative	3.1 (0.1)	233; 205; 190; 147; 131; 73	248.4668	[N]
11.162	3	Glycerol, 3TMS derivative	1.3 (0.1)	205; 147; 117; 103; 73	308.6372	[N]
12.145	4	Methylmalonic acid, 2TMS derivative	1.3 (0.1)	147; 73	262.4503	[N]
13.003	5	Glyceric acid, 3TMS derivative	1.0 (0.1)	292; 205; 189; 147; 103; 73	322.6207	[N]
15.093	6	Glutaric acid (Pentanedioic acid), 2TMS derivative	0.9 (0.1)	261; 204; 186; 158; 147; 73	276.4769	[N]
15.683	7	Butanoic acid, 2,4-bis[(trimethylsilyl)oxy]-, trimethylsilyl ester	2.0 (0.2)	321; 219; 147; 103; 73	336.6473	[N]
16.357	8	Butanoic acid, 3,4-bis[(trimethylsilyl)oxy]-, trimethylsilyl ester	1.5 (0.1)	321; 233; 189; 147; 117; 73	336.6473	[N]
17.308	9	Aminomalonic acid, tris(trimethylsilyl)-	0.8 (0.1)	320; 292; 218; 147; 73	335.6195	[N]
18.085	10	Malic acid, 3TMS derivative	1.8 (0.1)	335; 307; 245; 233; 189; 147; 73	350.6308	[N]
18.665	11	Unidentified	3.2 (0.2)	335; 293; 233; 217; 142; 71	–	–
20.613	12	Pentanedioic acid, 2-[(trimethylsilyl)oxy]-, bis(trimethylsilyl) ester	2.3 (0.2)	349; 247; 203; 147; 129; 73	364.6574	[N]
22.378	13	Unidentified	2.9 (0.1)	363; 261; 217; 171; 147; 129; 72	–	–
22.477	14	Unidentified	2.4 (0.1)	321; 305; 231; 147; 103; 73	–	–
22.588	15	Unidentified	3.5 (0.2)	430; 415; 355; 341; 281; 223; 147; 73	–	–
23.438	16	Unidentified	1.4 (0.1)	245; 231; 189; 147; 117; 73	–	–
27.993	17	Unidentified	0.8 (0.1)	189; 171; 147; 129; 73	–	–
28.637	18	Unidentified	3.7 (0.2)	347; 305; 243; 205; 189; 147; 117	–	–
28.925	19	Unidentified	2.3 (0.1)	347; 305; 243; 205; 189; 147; 117; 73	–	–
29.253	20	Unidentified	2.3 (0.03)	361; 347; 292; 257; 148; 73	–	–
29.843	21	Unidentified	2.3 (0.02)	361; 347; 292; 257; 221; 191; 147; 73	–	–
32.067	22	Palmitic Acid, TMS derivative	1.1 (0.04)	313; 145; 132; 129; 117; 73	328.6052	[N]
35.292	23	Unidentified	2.1 (0.1)	355; 341; 281; 221; 147; 73	–	–
42.795	25	β-D-Xylopyranose, 4TMS derivative	0.7 (0.1)	204; 147; 103; 73	438.8544	[N]
45.11	26	Unidentified	0.9 (0.01)	375; 285; 257; 204; 191; 147; 73	–	–
46.205	27	Unidentified	0.8 (0.04)	369; 281; 221; 147; 73	–	–
46.798	28	Unidentified	0.6 (0.003)	259; 204; 147; 103; 73	–	–
48.23	29	Unidentified	0.4 (0.02)	361; 204; 147; 103; 73	–	–
identified/ overall			17.9/ 47.8			
